# TRAF1 is a critical regulator of cerebral ischaemia–reperfusion injury and neuronal death

**DOI:** 10.1038/ncomms3852

**Published:** 2013-11-28

**Authors:** Yan-Yun Lu, Zuo-Zhi Li, Ding-Sheng Jiang, Lang Wang, Yan Zhang, Ke Chen, Xiao-Fei Zhang, Yi Liu, Guo-Chang Fan, Yingjie Chen, Qinglin Yang, Yan Zhou, Xiao-Dong Zhang, De-Pei Liu, Hongliang Li

**Affiliations:** 1 Department of Cardiology, Renmin Hospital of Wuhan University, Wuhan 430060, China; 2 Cardiovascular Research Institute of Wuhan University, Wuhan 430060, China; 3 State Key Laboratory of Medical Molecular Biology, Department of Biochemistry and Molecular Biology, Institute of Basic Medical Sciences, Chinese Academy of Medical Sciences & Peking Union Medical College, Beijing 100005, China; 4 College of Life Sciences, Wuhan University, Wuhan 430072, China; 5 Department of Pharmacology and Cell Biophysics, University of Cincinnati, Cincinnati, Ohio 45267-0575, USA; 6 Cardiovascular Division, University of Minnesota, Minneapolis, Minnesota 55455, USA; 7 Department of Nutrition Sciences, University of Alabama at Birmingham, Birmingham, Alabama 35294-3360, USA; 8 These authors contributed equally to this work

## Abstract

Stroke is a leading global cause of mortality and disability. Less than 5% of patients are able to receive tissue plasminogen activator thrombolysis within the necessary timeframe. Focusing on the process of neuronal apoptosis in the penumbra, which lasts from hours to days after ischaemia, appears to be promising. Here we report that tumour necrosis factor receptor-associated factor 1 (TRAF1) expression is markedly induced in wild-type mice 6 h after stroke onset. Using genetic approaches, we demonstrate that increased neuronal TRAF1 leads to elevated neuronal death and enlarged ischaemic lesions, whereas TRAF1 deficiency is neuroprotective. In addition, TRAF1-mediated neuroapoptosis correlates with the activation of the JNK pro-death pathway and inhibition of the Akt cell survival pathway. Finally, TRAF1 is found to exert pro-apoptotic effects via direct interaction with ASK1. Thus, ASK1 positively and negatively regulates the JNK and Akt signalling pathways, respectively. Targeting the TRAF1/ASK1 pathway may provide feasible therapies for stroke long after onset.

## Abstract (web summary)

TRAF1 is an intracellular signalling molecule that has diverse biological functions. In this study, the authors show that TRAF1 is expressed in mice soon after they have suffered a stroke and that increased TRAF1 expression increases susceptibility to ischaemia-induced apoptosis and brain injury.

## Graphical abstract

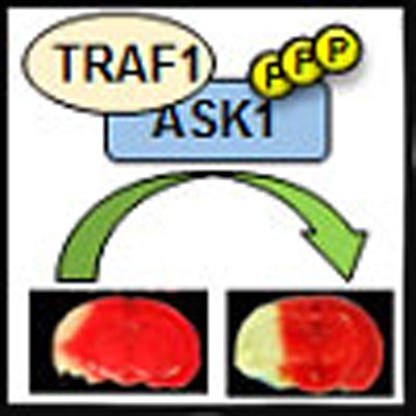

Stroke is a major global cause of death and permanent disability. Unfortunately, current therapeutic procedures are often limited to tissue plasminogen activator (tPA), which, 26 years after its introduction, remains the only US Food and Drug Administration (FDA)-approved treatment for rescuing brain tissue. A narrow 4.5 h therapeutic window has been imposed because of concerns regarding haemorrhagic complications; thus, tPA thrombolysis is procurable for <5% of stroke patients. Targeting molecular events during the subacute phase of stroke (hours–days) may provide a longer therapeutic window and thus greater clinical success^1^. Apoptosis is one of the fundamental mechanisms of cell death that occur during ischaemic brain injury^2^, and neurons are particularly vulnerable because of their high metabolic demand^3^. Furthermore, upon ischaemic stress, they exhibit biological hallmarks of apoptosis, including activation of pro-apoptotic bax, cytochrome c, caspase-9 and the effector caspase-3 as well as inhibition of anti-apoptotic bcl-2 family activity^2^,^4^. Targeting upstream death and survival signalling pathways, such as JNK and Akt, respectively, may ensure neuroprotective efficacy^5^,^6^. In a rodent stroke model, pharmacological inhibition of JNK, even when administered 6 h after cerebral ischaemia/reperfusion (I/R), was sufficient to reduce infarct size^7^. Furthermore, suppression of CTMP, an inhibitor of the pro-survival Akt, ameliorated neuronal injury and cognitive deficits^6^. However, the underlying mechanisms integrating these pathways are not clear.

The TRAF family includes seven members (TRAF1-7) and connects the tumour-necrosis factor receptor (TNFR), the Toll-like receptor (TLR) and the interleukin-1 (IL-1) superfamily in signalling cascades. TRAF1 and TRAF2 were the first members of this family to be identified and recognized as adaptor proteins of TNFR2 (ref. 8). Compared with other TRAFs, TRAF1 expression is low in resting cells and is restricted to the spleen, lung and testis in mice and humans^8^,^9^. Recent studies have shown that TRAF1 expression can be greatly induced by several distinct types of stimuli^10^ and is dysregulated in diseases such as atheromas^11^, lymphomas^12^ and solid tumors^13^. TRAF1 interacts with multiple receptors, kinases, adaptors and regulator proteins, contributing to its diverse biological functions^14^. However, the role of TRAF1 in apoptosis remains unclear; Wang *et al*.^15^ demonstrated that TRAF1 exerts an anti-apoptotic effect, whereas Jang *et al*.^16^ reported that TRAF1 can be cleaved and consequently enhance Fas ligand- or TNF-α-induced apoptosis. Moreover, TRAF1 sustains the activation of pro-apoptotic JNK mediated by TRAF2 (ref. 17). In a recent study on renal cell carcinoma, TRAF1 inhibition resulted in a decreased apoptotic rate in tumour cells^13^. Furthermore, regulation of cell survival, proliferation, differentiation and death by TRAFs has been studied extensively; these studies have indicated a potential role of these adaptor proteins in the susceptibility of tissues and organs to stress-induced injury, although knowledge regarding the biological roles of TRAF1 in neurological systems remains rudimentary.

In the present study, we observed a robust induction of TRAF1 expression in mouse brain neurons 6 h after a stroke. Accordingly, sustained TRAF1 overexpression in neurons led to increased susceptibility to ischaemia-induced apoptosis and brain injury, whereas loss of TRAF1 expression was neuroprotective. Mechanistically, TRAF1-mediated neuroapoptosis was regulated by the activation of the JNK pro-death pathway and inhibition of the Akt cell survival pathway. Thus, TRAF1 has a critical role in neuronal cell fate in response to I/R, offering a novel therapeutic target for stroke treatment with a relatively longer timeframe of use.

## Results

### Neuronal TRAF1 expression is upregulated after stroke

**Figure 1:**
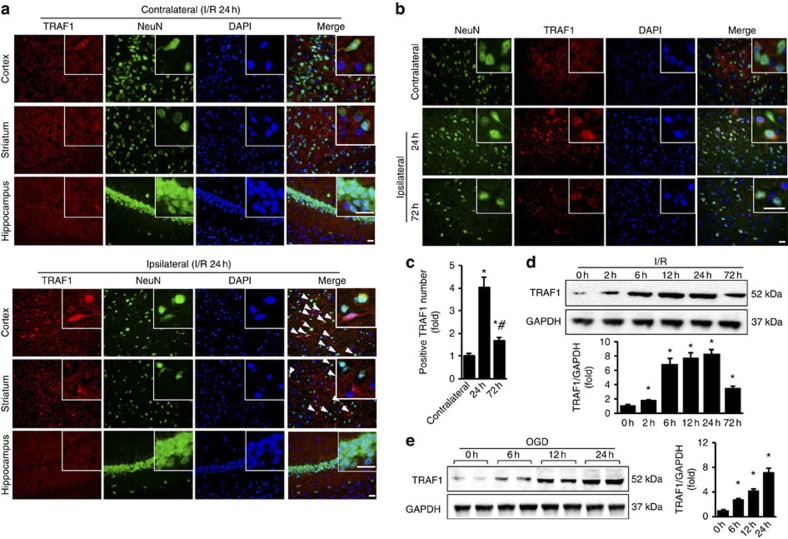
TRAF1 in neurons is upregulated with stroke. (**a**) Co-staining of TRAF1 (red), NeuN (green) and nuclei (blue) demonstrates TRAF1 upregulation (arrowheads) in neurons of the ipsilateral (lower) hemisphere compared with the contralateral (upper) hemisphere 24 h after of ischaemia/reperfusion (I/R). Insets show a higher magnification view. Scale bar, 20 μm. (**b**) Representative images of cortices co-stained for TRAF1 (red), NeuN (green) and nuclei (DAPI, blue) after 24 or 72 h of I/R. Insets show a higher magnification view. Scale bar, 20 μm. (**c**) Quantification of TRAF1–NeuN co-immunostaining at the indicated time points (*n*=4, *P<0.05 versus the contralateral cortex; ^#^*P*<0.05 versus the ipsilateral cortex after 24 h of I/R). (**d**) Immunoblots showing TRAF1 expression at the indicated time points following ischaemic onset (top). GAPDH served as a loading control. Bottom panel: quantification of normalized TRAF1 levels (*n*=6 per time point, *P<0.05 versus sham). (**e**) Immunoblots of TRAF1 in extracts of OGD-treated neurons (left) and quantification of TRAF1 levels (right, *n*=6, *P<0.05 versus control). The data represent the mean±s.d. Statistical analysis was carried out by unpaired Student’s *t*-test.

We generated an experimental stroke model induced by middle cerebral artery occlusion (MCAO) for 1 h followed by various periods of reperfusion. Immunofluorescence staining revealed increased TRAF1 signals in the peri-infarct area (ipsilateral cortex) compared with the contralateral control following I/R; these increases were observed primarily in the cortex and stratum but not in the hippocampus (Fig. 1a). Neurons are more vulnerable to stroke insult than other cells in the brain^2^. Double-immunofluorescence labelling detected a robust increase in cytoplasmic TRAF1 expression in cortical neurons (Fig. 1b) that peaked 24 h after ischaemic onset at ~4.0-fold the normal level and then dropped at 72 h to ~1.7-fold (Fig. 1c). Western blot analysis of ipsilateral brain homogenates revealed a similar TRAF1 expression pattern (Fig. 1d).

To determine whether neuronal TRAF1 is functionally upregulated, we cultured primary neurons from the rat cortex; challenged them for 1 h with oxygen and glucose deprivation (OGD), which mimics the conditions of low oxygen and low glucose during vascular stroke injury; and returned the cultures to normal conditions for various periods of time (Supplementary Fig. S1a). Double-immunofluorescence labelling of TRAF1 and MAP2 demonstrated that OGD increased TRAF1 expression in a time-dependent manner (Supplementary Fig. S1b), and our western blot results confirmed that neuronal TRAF1 was upregulated after OGD to a maximum of ~7.2-fold greater than that in the controls (Fig. 1e). Collectively, these data indicate that TRAF1 is dramatically upregulated in neurons following I/R. Because of the low TRAF1 expression in normal brain and resting cells^8^,^9^, we further investigated whether this prominent upregulation of neuronal TRAF1 expression impacts ischaemia-induced neuronal and cerebral damage.

### TRAF1 transgenic mice exhibit enlarged stroke lesions

To elucidate the direct effects of upregulated neuronal TRAF1 expression on stroke outcome, we generated several lines of neuron-specific *Traf1* transgenic (TG-TRAF1) mice. TRAF1 expression in neurons is driven by the platelet-derived growth factor promoter (Supplementary Fig. S2a), and the genotypes of the TG-TRAF1 mice were confirmed through PCR (Supplementary Fig. S2b). To reflect physiological conditions during stroke, TG2-TRAF1 mice, which exhibit levels of cerebral TRAF1 expression similar to those induced by MCAO *in vivo* (Supplementary Fig. S2c), were used for further experiments. In addition, all mouse strains were normal in appearance and fertile. To evaluate whether TRAF1 neuron-specific overexpression resulted in phenotypic changes to the cerebral vasculature, we inspected the Circle of Willis, anterior cerebral arteries, middle cerebral arteries (MCAs) and posterior cerebral arteries of the TG2-TRAF1 and non-transgenic (NTG) mice using India ink staining. We did not observe gross anatomical differences that might affect stroke outcome (Supplementary Fig. S2d), so to eliminate the possibility that TRAF1 might have altered cerebral perfusion and impacted infarct volumes, regional cerebral blood flow (rCBF) was monitored by Doppler flowmetry throughout the MCAO procedure. TG2-TRAF1 mice exhibited no significant difference in rCBF compared with NTG mice at baseline (Supplementary Fig. S2e). Ligation of the common carotid artery resulted in a comparable reduction in blood flow in both experimental groups, and rCBF in the MCA territory returned to baseline levels after reperfusion (Supplementary Fig. S2e). Because both hypertension and arrhythmia are particularly notable stroke risk factors^2^, we then measured blood pressure and heart rate in both strains; however, no differences in systolic blood pressure, diastolic blood pressure or heart rate were observed between the groups (Supplementary Table S1); and furthermore, no significant changes were noted in blood gases, pH or body weight.

**Figure 2:**
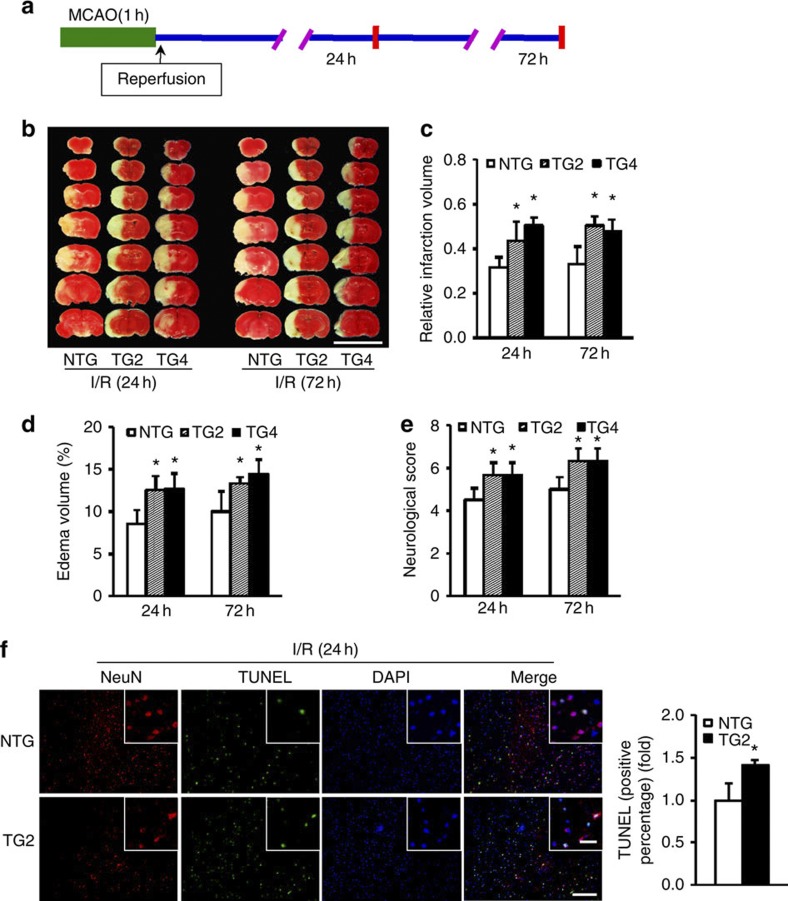
TRAF1 neuron-specific transgenic mice have enlarged brain lesions after stroke. (**a**) Time course of MCAO and reperfusion before sacrifice. (**b**) Brains of NTG, TG2-TRAF1 and TG4-TRAF1 mice stained with TTC at the indicated times after I/R. Scale bar, 10 mm. (**c**–**e**) Quantification of infarct (**c**), oedema (**d**) volumes and neurological scores (**e**) at the indicated times (*n*=7 at each time point, *P<0.05 versus NTG). (**f**) Representative images of apoptosis in cortical neurons co-stained with NeuN and TUNEL after 24 h of reperfusion (left). Scale bar, 100 μm. Insets show a higher magnification view. Scale bar, 20 μm. The right panel shows the quantification of TUNEL-positive cells (*n*=4, *P<0.05 versus NTG). The data represent the mean±s.d. Statistical analysis was carried out by unpaired Student’s *t*-test.

TG2-TRAF1, TG4-TRAF1 and NTG mice were challenged with tMCAO for 60 min and examined for 2,3,5-triphenyl-2H-tetrazolium chloride (TTC) staining after 23 or 71 h of reperfusion (Fig. 2a,b). Compared with their NTG siblings, a 6.7-fold increase in TRAF1 expression in TG2-TRAF1 mice enlarged the infarct volume by ~37% and 53% (Fig. 2c) and the oedema volume by ~47% and 34% (Fig. 2d) at 24 and 72 h, respectively, after MCAO. TG2-TRAF1 mice also had significantly higher neurological scores at both time points, with increases of ~26% and 27% at 24 and 72 h, respectively (Fig. 2e). TG4-TRAF1 mice displayed an ~9.2-fold increase in TRAF1 expression compared with NTG mice and displayed outcomes similar to TG2-TRAF1 mice (Fig. 2c–e). Notably, no significant change in cerebral TRAF1 expressions was observed in both TG2 and TG4 mice before and after I/R insults (Supplementary Fig. S2f). Neurons have limited ability to regenerate and are particularly at risk for programmed cell death due to their high metabolic demand during ischaemic brain injury^3^. Thus, we evaluated neuroapoptosis by performing TUNEL assays and NeuN dual-immunofluorescence staining 24 h after transient focal ischaemia and found an increase of ~41% in the apoptotic rate in TG2-TRAF1 mice compared with that in NTG mice (Fig. 2f). Thus, these data demonstrate that neuronal TRAF1 has a detrimental effect on both histological cerebral ischaemic injury and behavioural neurological dysfunction, which is most likely attributable to the enhancement of delayed neuronal apoptosis.

### TRAF1-deficient mice have reduced stroke lesions

**Figure 3:**
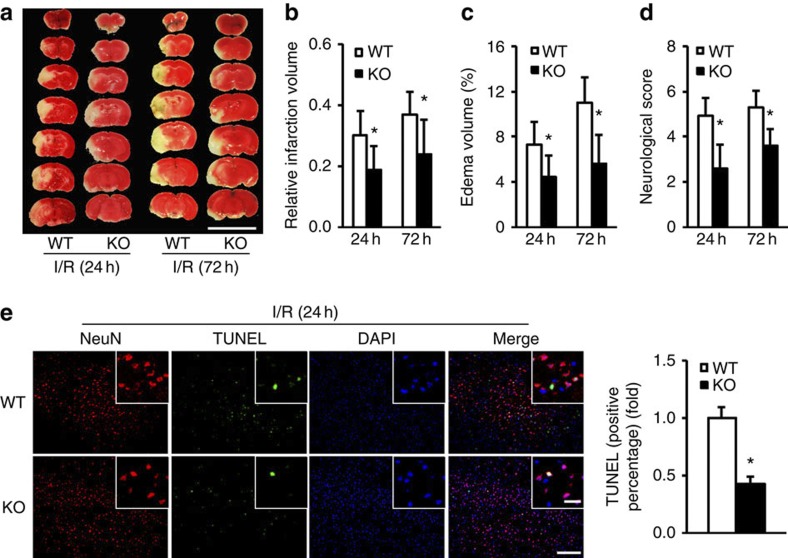
TRAF1 deficiency is neuroprotective during stroke. (**a**) Brains stained with TTC at the indicated times after ischaemia/reperfusion (I/R). Scale bar, 10 mm. (**b**–**d**) Quantification of infarct (**b**) and oedema (**c**) volumes and neurological scores (**d**) at the indicated times (*n*=7 or 12 at each time point, *P<0.05 versus WT). (**e**) Representative images of apoptosis in cortical neurons of WT and TRAF1-KO mice co-stained with NeuN and TUNEL after 24 h of I/R (left). Scale bar, 100 μm. The inset shows a higher magnification view. Scale bar, 20 μm. The right panel shows quantification of TUNEL-positive cells (*n*=4, *P<0.05 versus NTG). The data represent the mean±s.d. Statistical analysis was carried out by unpaired Student’s *t*-test.

We hypothesized that if TRAF1 has a detrimental role during stroke, then inhibiting the post-stroke generation of TRAF1 in *Traf1* knockout mice (TRAF1-KO) might prevent neuronal damage. All mouse strains were generated in agreement with predicted Mendelian ratios (85 wild-type (WT), 152 heterozygous and 81 TRAF1-KO mice). As expected, the TRAF1 protein was not expressed in the brains of TRAF1-KO mice before or after MCAO (Supplementary Fig. S3a). Likewise, we ruled out potential changes in gross neurovascular anatomy (Supplementary Fig. S3b), rCBF (Supplementary Fig. S3c) and other physiological parameters, including blood pressure, blood gases, pH and body weight (Supplementary Table S1). Importantly, TRAF1 deficiency led to ~38% and 39% reductions in infarct and oedema volumes, respectively, compared with those in WT mice 24 h after MCAO (Fig. 3a–c). These neuroprotective effects on the infarct and oedema volumes persisted until 71 h after reperfusion (35% and 49% reductions, respectively, compared with WT; Fig. 3b,c). In addition, the neurological score was mitigated by >30% in TRAF1-KO mice at both time points (Fig. 3d). Consistent with these observations, TRAF1-KO mice displayed an ~57% reduction in TUNEL-positive cells 24 h after I/R (Fig. 3e), further indicating a critical role of TRAF1 in ischaemic brain injury. Therefore, inhibiting TRAF1 induction after cerebral ischaemia could be neuroprotective.

### TRAF1 promotes ischaemia-induced neuronal apoptosis

**Figure 4:**
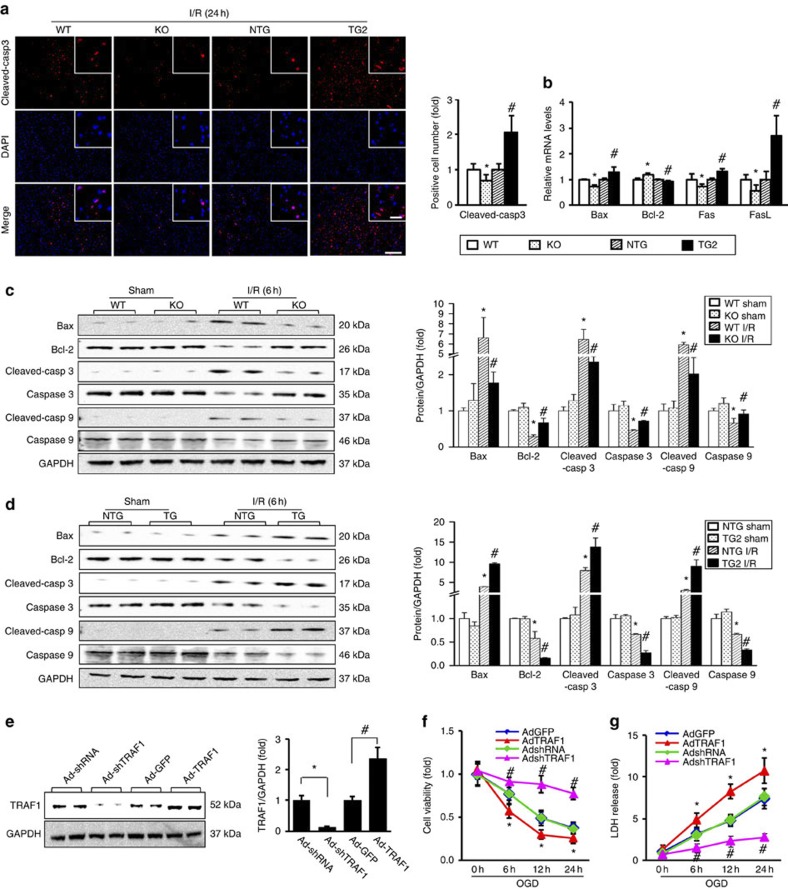
TRAF1 modulates neuronal apoptosis in ischaemic brain. (**a**) Fluorescence staining of cleaved caspase-3 (red) and nuclei (DAPI, blue) in the brains of WT, TRAF1-KO, NTG, and TG2-TRAF1 mice after 24 h of I/R. Scale bar, 100 μm. The inset shows a higher magnification view. Scale bar, 20 μm. The right panel shows the quantification of immunopositive cells (*n*=3–4, *P<0.05 versus WT, ^#^*P*<0.05 versus NTG). (**b**) Quantitation of the indicated mRNA levels (*n*=4, *P<0.05 versus WT, ^#^*P*<0.05 versus NTG). (**c**,**d**) Immunoanalysis of pro-apoptotic proteins in the brain extracts of WT and TRAF1-KO (**c**) and TG2-TRAF1 and NTG (**d**) mice 6 h after sham surgery or I/R. The right panels show the quantification of normalized protein levels (*n*=6, *P<0.05 versus sham-operated WT (**c**) or NTG (**d**); ^#^*P*<0.05 versus I/R-operated WT (**c**) or NTG (**d**)). (**e**) Immunoanalysis of TRAF1 in neurons infected with the indicated adenoviral vectors (left) and quantification of TRAF1 expression normalized to GAPDH (right) (*n*=6, *P<0.05 versus Ad-shRNA, ^#^*P*<0.05 versus Ad-GFP). (**f**,**g**) Cell viability (**f**) and LDH release (**g**) after adenoviral infection of neurons challenged by OGD for the indicated times (*n*=6 for each time point, *P<0.05 versus Ad-GFP, ^#^*P*<0.05 versus Ad-shRNA). The data represent the mean±s.d. Statistical analysis was carried out by unpaired Student’s *t*-test.

Given that neuronal apoptosis has been suggested to underlie progressive neuropathology and neurological dysfunction hours or days post-stroke^18^, we investigated the biological role of TRAF1 in neuroapoptotic cascades *in vivo*. We first examined caspase-3, which is a pivotal effector of apoptosis in ischaemic stroke^4^, and found that the number of cleaved (active) caspase-3-positive cells was ~31% lower in TRAF1-KO mice and ~2.1-fold greater in TG2-TRAF1 mice than in controls (Fig. 4a). TRAF1 deficiency also upregulated the mRNA and protein expression levels of Bcl2 (a neuroprotective, anti-apoptotic gene^4^), whereas TRAF1 deficiency suppressed the expression of the pro-apoptotic genes Bax, Fas and FasL (the last two are crucial mediators of the extrinsic mechanism^4^) 6 h after MCAO (Fig. 4b–d). Thus, these results suggest that TRAF1 is an upstream modulator of the mitochondrial apoptotic pathway in neurons. To further validate that TRAF1 accelerates programmed neuronal death, we investigated whether TRAF1 directly affects neuronal survival in cultured cortical neurons. We performed gain- and loss-of-function experiments using an adenovirus harbouring human TRAF1 cDNA (Ad-TRAF1) or TRAF1 short hairpin RNA (Ad-shTRAF1). Under basal conditions, no significant difference in neuronal survival was observed between the treatments. However, when challenged with OGD, Ad-TRAF1 infection, which increased TRAF1 expression by ~2.4-fold (Fig. 4e), led to fewer viable neurons (Fig. 4f) and increased lactate dehydrogenase (LDH) release (Fig. 4g) at the different time points. By contrast, Ad-shTRAF1 treatment, which decreased TRAF1 expression by ~88% (Fig. 4e), significantly counteracted OGD-induced cell death (Fig. 4f,g). Consistent with these results, the positive and negative regulation of pro- and anti-apoptotic proteins, respectively, by TRAF1 in OGD-challenged cells (Supplementary Fig. S4a,b) was similar to that observed *in vivo*. Therefore, our results show that TRAF1-mediated ischaemic cerebral injury is, at least in part, attributable to delayed neuronal cell death. Thus, targeting TRAF1 may render neurons resistant to programmed cell death following ischaemia–reperfusion.

### TRAF1 promotes ischaemic brain injury via ASK1 activation

**Figure 5:**
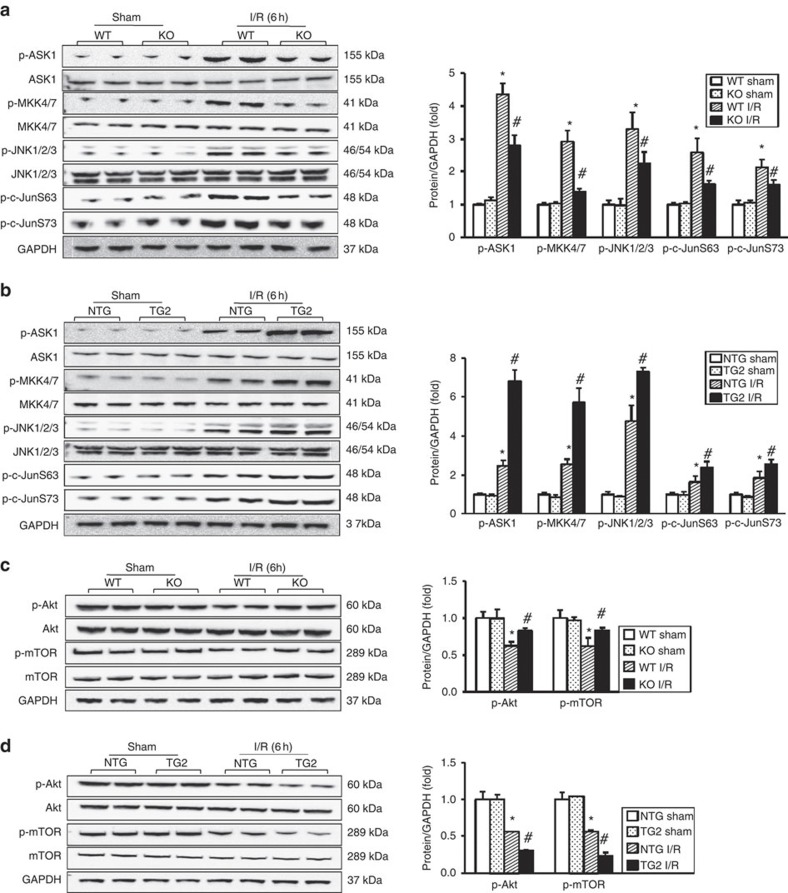
TRAF1 activates ASK1 signalling pathways. (**a**,**b**) TRAF1 activates the ASK1/JNK pathway. Western analysis (left) and quantification (right) of the levels of the indicated proteins in the brains of (**a**) TRAF1-KO, WT and (**b**) TG2-TRAF1, NTG mice 6 h after sham surgery or ischaemia/reperfusion (I/R) (*n*=6, *P<0.05 and ^#^*P*<0.05 versus WT and NTG with and without I/R, respectively). (**c**,**d**) TRAF1 suppresses the Akt cell survival pathway. Analysis of the indicated proteins in mice under the same experimental conditions described in **a** and **b**, respectively (*n*=6). The data represent the mean±s.d. Statistical analysis was carried out by unpaired Student’s *t*-test.

On the basis of the observation that TRAF1 activates the Fas/FasL apoptotic pathway, we proposed that TRAF1 functions upstream of the mitochondria-mediated apoptosis signalling pathway. The MAPK family, which consists of p38 MAPK, ERK and JNK, is increasingly being recognized for its involvement in controlling post-ischaemic neuronal cell death^1^. Therefore, we investigated whether these pathways are modulated by TRAF1 upon ischaemic insults. TRAF1 exerted no significant effect on either the expression or phosphorylation of ERK and p38 (Supplementary Fig. S5a,b). JNK, which has been shown to be markedly upregulated 6 h after cerebral ischaemic injury in an experimental stroke model^19^, is known to mediate the induction of a series of pro-apoptotic proteins, including c-Jun, Bim, Bax and Fas, upon I/R^20^. In our model, TRAF1-KO mice displayed reduced phosphorylation of JNK and its major downstream molecule, c-Jun, at serines 63 and 73 (Fig. 5a). Because JNK is activated by a number of upstream stress-related activators, including ASK1 and MKK4/7 (ref. 21), we examined whether ASK1 activation was altered; we observed an ~36% decrease in the expression of active, phosphorylated ASK1 (p-ASK1), concurrent with inhibition of the MKK/JNK pathway (Fig. 5a). Conversely, we found that TRAF1 overexpression increased p-ASK1 expression to ~1.76-fold greater than that in NTG controls and enhanced the activation of the MKK/JNK pathway (Fig. 5b), thus aggravating I/R-induced cerebral injury. To verify the positive regulation of the ASK1/JNK pathway by TRAF1, we used gain- and loss-of-function approaches. Western blot analysis confirmed that ASK1/JNK activation was positively regulated by TRAF1 upon OGD stress *in vitro* (Supplementary Fig. S6). Notably, inhibition of TRAF2 did not interfere with TRAF1-mediated ASK1 phosphorylation during OGD/reperfusion (Supplementary Fig. S7). Thus, TRAF1 may underlie, at least in part, post-stroke neuroapoptosis through the ASK1/JNK pathway. Another crucial signalling pathway involved in I/R upstream of the mitochondrial cascade is the Akt pathway. Akt promotes cell survival via phosphorylation and activation of the pro-survival mTOR and CREB pathways^22^. Furthermore, Subramanyam *et al*.^23^ observed that ASK1 inhibits Akt phosphorylation and activation and participates in the apoptotic inhibition of the regulatory volume increase in HeLa cells. However, whether Akt protects against neuronal death downstream of ASK1 is not known. We found that TRAF1-induced ASK1 activation coincided with reduced phosphorylation of Akt and the downstream molecule mTOR both *in vivo* (Fig. 5c,d) and *in vitro* (Supplementary Fig. S8a,b). The activity of the Akt/mTOR signalling pathway was significantly enhanced in the brains of TRAF1-KO mice after MCAO as well as in neurons infected with Ad-shTRAF1 and exposed to OGD. Akt phosphorylates CREB, leading to CREB-mediated expression of genes crucial for neuronal survival, including brain-derived neurotrophic factor (BDNF). The CREB/BDNF pathway was activated in TRAF1-KO mice following MCAO, resulting in increased expression of the pro-survival factors GAP43 and NGF (Supplementary Fig. S9a). Conversely, TG2-TRAF1 suppressed CREB and BDNF phosphorylation and decreased GAP43 levels (Supplementary Fig. S9b). Together, these findings indicate that TRAF1 confers pro-apoptotic functions via regulation of both the JNK and Akt pathways and that this regulation is most likely mediated by ASK1 activation.

### TRAF1 directly interacts with ASK1

**Figure 6:**
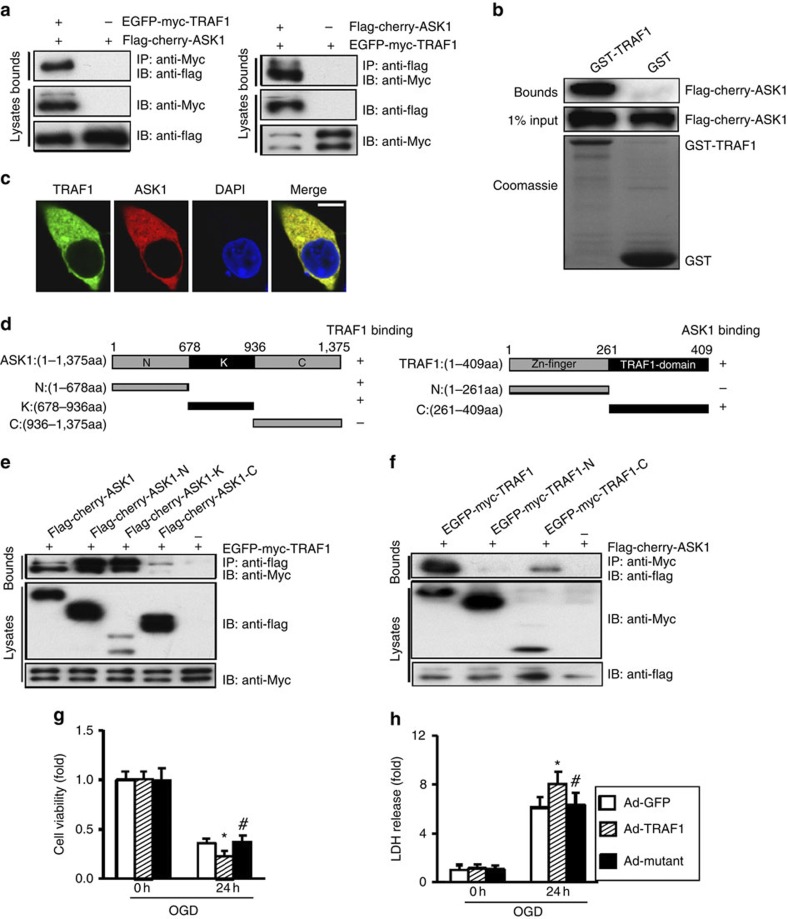
TRAF1 directly interacts with ASK1. (**a**) Co-IP of TRAF1 and ASK1. HEK293T cells were transfected with Myc-tagged TRAF1 and FLAG-tagged ASK1. The lysates were immunoprecipitated with anti-Myc or anti-FLAG and analysed by immunoblotting using anti-Myc or anti-FLAG antibodies. (**b**) Immunoanalysis of GST pull-down of FLAG-tagged ASK1 with GST-TRAF1 or GST. (**c**) Co-localization of TRAF1 and ASK1 in the cytoplasm of HEK293T cells; the nuclei were stained with DAPI. Scale bar, 5 μm. (**d**) Schematic representation of ASK1 and TRAF1 deletion mutants. (**e**) Mapping of the TRAF1-binding region of ASK1. HEK293T cells were transfected with Myc-tagged TRAF1, and FLAG-tagged ASK1 deletion mutants were immunoprecipitated from lysates with anti-FLAG and immunoblotted with anti-Flag and anti-Myc. (**f**) Mapping of the ASK1 binding region of TRAF1. HEK293T cells were transfected with FLAG-tagged ASK1, and the lysates were immunoprecipitated with anti-Myc and immunoblotted with anti-Flag and anti-Myc. (**g**,**h**) TRAF1-ASK1 interaction is required for TRAF1-mediated neuronal injury. Cultured neurons were infected with Ad-GFP, Ad-TRAF1 or Ad-mutant harbouring a mutant TRAF domain and subject to OGD for the indicated times. The Ad-mutant completely rescued the Ad-TRAF1-mediated decrease in cell survival (**g**) and increase in LDH release (**h**) (*n*=9, *P<0.05 versus Ad-GFP; ^#^*P*<0.05 versus Ad-TRAF1). The data represent the mean±s.d. Statistical analysis was carried out by unpaired Student’s *t*-test.

Next, we examined whether ASK1 is a direct target of TRAF1. We found that in HEK293T cells transfected with Myc-tagged TRAF1 and Flag-tagged ASK1, TRAF1 co-immunoprecipitated with ASK1, and vice versa (Fig. 6a). The results of our glutathione *S*-transferase (GST) pull-down assay (Fig. 6b) and the endogenous co-immunoprecipitation (co-IP) performed in primary cortical neurons (Supplementary Fig. S10) further supported the finding that TRAF1 interacts with ASK1. Next, we investigated TRAF1 and ASK1 localization and observed that these proteins were predominantly co-localized in the cytoplasm (Fig. 6c). To identify the protein domains critical for TRAF1 and ASK1 association, we generated a series of truncated Flag-tagged ASK1 constructs (Fig. 6d) and co-transfected ASK1 deletion mutants with Myc-tagged TRAF1 followed by co-IP. The results reproducibly demonstrated that either the N-terminal region or kinase domain of ASK1 but not the C-terminal region retained the ability to interact with TRAF1 (Fig. 6e). TRAF1 comprises a C-terminal (TRAF) domain responsible for interactions with receptors and other signalling proteins^24^ and an N-terminal domain that lacks the RING finger domain present in other TRAFs. Using TRAF-domain and N-terminal-domain truncation and deletion mutants (Fig. 6d), we found that only the TRAF domain bound to ASK1 (Fig. 6f). To determine whether this binding domain directly mediates TRAF1-induced ischaemic neuronal injury, we generated an adenoviral vector encoding TRAF1 with a mutant TRAF domain (Ad-mutant). Indeed, 24 h after OGD treatment, cultured neurons infected with Ad-TRAF1, but not those infected with Ad-mutant, displayed a reduction in cell viability of ~37% (Fig. 6g) and an increase in LDH release by 32% (Fig. 6h) compared with the Ad-GFP control. Therefore, the TRAF domain is required for TRAF1-mediated neuronal death following ischaemia. Notably, simple genetic TRAF1 manipulations failed to impact ASK1 activation in sham-operated mice (Fig. 5a,b) and control neuronal cultures (Supplementary Fig. S6a,b). We thus speculated that I/R exposure is necessary for TRAF1-ASK1 interaction and, subsequently, ASK1 phosphorylation. To test this hypothesis, we isolated primary cortical neurons and infected them with Ad-TRAF1 prior to OGD exposure. The protein extract was immunoprecipitated with anti-TRAF1 antibodies and immunoblotted with anti-ASK1 antibodies, and vice versa (Supplementary Fig. S11). Intriguingly, although the TRAF1-ASK1 interaction was relatively weak in normal neuronal cultures, TRAF1 readily bound to ASK1 after an OGD challenge (Supplementary Fig. S11). Therefore, our results indicate that in response to I/R, TRAF1 expression is induced in neurons, and the ability of TRAF1 to interact with ASK1 is improved.

### TRAF1-induced neuronal injury is largely ASK1-dependent

**Figure 7:**
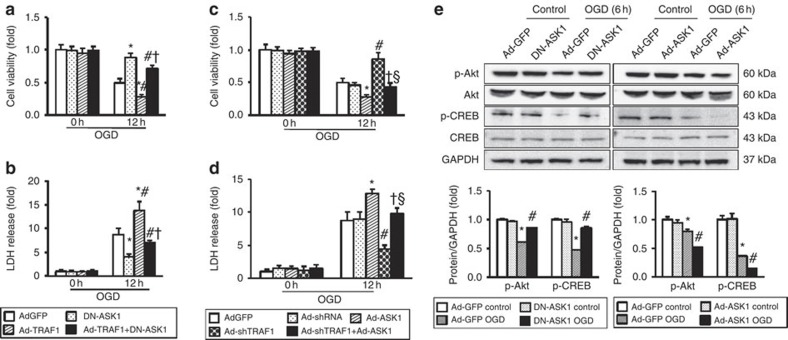
TRAF1-mediated neuronal damage requires activation of ASK1 signalling cascades. (**a**–**d**) TRAF1-mediated neuronal cell death is ASK1-dependent. (**a**,**b**) Cultured cortical neurons were infected with adenovirus encoding TRAF1 and dominant negative ASK1 (DN-ASK1), alone or together, and subjected to OGD for the indicated times. Cell viability (**a**) and LDH release (**b**) were assayed (*n*=6, *P<0.05 versus Ad-GFP, ^#^*P*<0.05 versus DN-ASK1, ^†^*P*<0.05 versus Ad-TRAF1). (**c**,**d**) Cultured cortical neurons were infected with adenovirus encoding short hairpin RNA targeting TRAF1 (Ad-shTRAF1) and ASK1, or both, and subjected to OGD for the indicated times. Cell viability (**c**) and LDH release (**d**) were assayed (*n*=6, *P<0.05 versus Ad-GFP, ^#^*P*<0.05 versus Ad-shRNA, **^†^***P*<0.05 versus Ad-ASK1, ^§^*P*<0.05 versus Ad-shTRAF1). (**e**) The effect of ASK1 on the Akt pathway upon ischaemia/reperfusion. Cultured cortical neurons were infected with Ad-ASK1 or DN-ASK1, with or without subsequent exposure to OGD for 6 h. Immunoanalysis (top) and quantification (bottom) of the indicated proteins in lysates are shown (*n*=6, *P<*P*<0.05 and ^#^*P*<0.05 versus Ad-GFP with and without OGD treatment, respectively). The data represent the mean±s.d. Statistical analysis was carried out by unpaired Student’s *t*-test.

To further investigate whether TRAF1-induced neuronal injury is ASK1-dependent, we generated adenoviral vectors encoding the WT (Ad-ASK1) and dominant negative (DN-ASK1) forms of ASK1 (ref. 25) and examined neuronal viability and LDH release. Under basal conditions, alterations in either TRAF1 or ASK1 expression had no significant effect on either parameter. When cells were exposed to OGD, however, co-infection with DN-ASK1 counteracted Ad-TRAF1-induced neuronal cell death (Fig. 7a) and reduced LDH release (Fig. 7b). Conversely, Ad-shTRAF1-induced neuroprotection was abolished by ectopic ASK1 expression (Fig. 7c,d). Thus, ASK1 is indispensable for TRAF1-mediated neuronal cell injury. To further characterize the role of ASK1 in neuronal survival, we infected cultured neurons with adenoviruses that encode gain (Ad-ASK1) or loss of (DN-ASK1) ASK1 and challenged the cells with OGD. Ectopic ASK1 expression recapitulated TRAF1 overexpression by diminishing Akt and CREB phosphorylation (Fig. 7e); this finding indicates that the Akt survival pathway was directly suppressed by ASK1. By contrast, DN-ASK1 elevated the expression of phosphorylated Akt and CREB. Therefore, TRAF1 modulates neuronal cell injury, at least in part, through direct regulation of ASK1-dependent pathways.

Because Fas/FasL is necessary for JNK-induced neuronal apoptosis and has been implicated in stroke^26^, we next evaluated whether Fas activation is involved in TRAF1/ASK1-mediated neuronal death. Primary cortical neurons were infected with AdTRAF1 or the AdGFP control and incubated with Fas–Fc (the Fc fragment of human IgG1 fused to the Fas ectodomain) or an anti-FasL antibody before OGD exposure. Fas–Fc and anti-FasL sufficiently salvaged neurons from OGD-induced cell death (Supplementary Fig. S12). Moreover, TRAF1-mediated neuronal death was completely abrogated when Fas–Fc and anti-FasL antibodies were employed (Supplementary Fig. S12), suggesting that the Fas/FasL pathway is a major TRAF1 target during ischaemic stroke. Because Fas might induce cell death through caspase-independent necroptosis, we then examined whether necroptosis is involved in the pro-death effect of TRAF1 (ref. 27). Primary neuronal cultures infected with either AdTRAF1 or AdGFP control were incubated with necrostatin-1 (an inhibitor of necroptosis) and Z-VAD.FMK (a pan-caspase inhibitor), separately or jointly^28^. Although Z-VAD.FMK significantly reduced neuronal death after OGD treatment, necrostatin-1 exhibited no effect on cell survival in our *in vitro* stroke model (Supplementary Fig. S13). Furthermore, Z-VAD.FMK, but not necrostatin-1, sufficiently protected neurons against TRAF1-mediated death (Supplementary Fig. S13). Together with our previous results, these findings demonstrate that TRAF1 targets the ASK1/JNK pro-death pathway and the Akt pro-survival pathway, which in turn induces Fas/FasL-associated neuronal apoptosis in ischaemic stroke.

## Discussion

**Figure 8:**
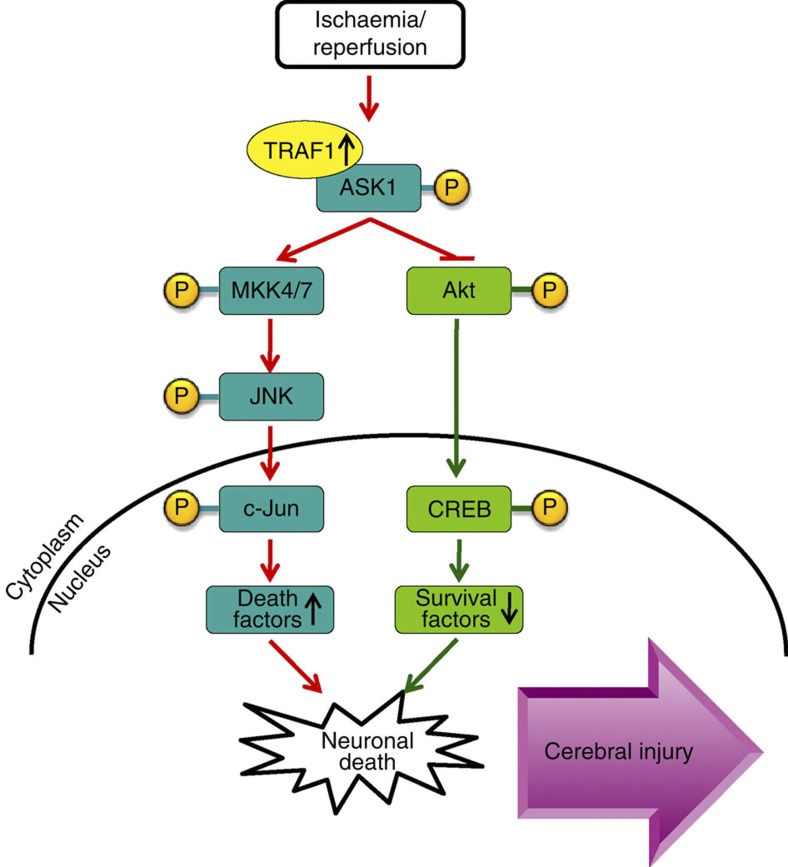
Proposed mechanism of TRAF1-mediated I/R cerebral injury. With ischaemia/reperfusion, upregulated TRAF1 facilitates ASK1 phosphorylation at Thr845 through a direct interaction. Activated ASK1 synergistically phosphorylates and activates the MKK/JNK pro-death pathway and inhibits the Akt pro-survival signalling pathway, leading to enhanced neuronal death and cerebral damage.

Despite extensive investigations of the mechanisms of ischaemic cerebral injury, little progress has been made in translating these findings into clinical practice. The current lack of appropriate therapies for stroke is, at least in part, a result of the insufficient therapeutic window for efficacy^29^. The present study provides exciting insight into the previously unidentified but essential roles of TRAF1 in late neuronal apoptosis, a primary pathophysiological process lasting hours to days post-stroke^18^, and the resulting brain damage. Several stages characterize TRAF1-mediated cerebral ischaemic injury. First, neuronal TRAF1 was robustly induced during the subacute phase of stroke. Second, the induced TRAF1 facilitated ASK1 phosphorylation and activation in neurons. Third, ASK1 promoted neuronal apoptosis by both activating the MKK/JNK cell death pathway and suppressing the Akt cell survival signalling pathway. Finally, these combined effects led to the deterioration of histological and behavioural stroke outcomes (Fig. 8). To the best of our knowledge, this study is the first demonstration that TRAF1 has a crucial role in mediating post-stroke neuronal death. More importantly, we showed that a genetic TRAF1 deletion that blocks aberrant TRAF1 upregulation after I/R resulted in a marked reduction in infarct size and improved the neurological outcome. Therefore, TRAF1 represents a promising and durable therapeutic target for treating ischaemic stroke.

Although TRAF1 was initially proposed to exhibit anti-apoptotic properties^15^, in this study, we have demonstrated that TRAF1 acts as a pro-apoptotic mediator in MCAO-induced ischaemic stroke. Consistent with our findings, recent studies have indicated a role for TRAF1 in promoting programmed cell death, although the underlying mechanisms remain to be elucidated^13^,^16^. The contradictory roles of TRAF1 in the regulation of cell survival may be attributable to the differences in experimental settings, cell types, stimuli and more importantly, target genes. We also showed that the interaction with ASK1 is essential for TRAF1-mediated neuroapoptosis. ASK1 is a major kinase activated by stress and has crucial roles in neuroapoptosis and ischaemic stroke^30^,^31^. On the basis of our findings, we summarized the mechanisms by which the TRAF1/ASK1 pathway contributes to brain ischaemic injury as follows: first, TRAF1 co-localizes with ASK1 in the cytoplasm and binds directly to the ASK1 N-terminal and kinase domains via its TRAF domain. Second, this interaction facilitates ASK1 phosphorylation and activation. Finally, activated ASK1 potentiates MKK/JNK and suppresses the Akt signalling pathway, thus synergistically promoting neuron death following ischaemic insults. In previous studies, TRAF1 has been referred to primarily as an adaptor protein that elaborates receptor signal transduction^32^; however, knowledge regarding its downstream signalling has been limited to its indirect regulation of NF-κB^17^,^33^. Our study identified ASK1 as a novel direct target of TRAF1 in the brain and potentially extends the biological functions of TRAF1 to other neurodegenerative diseases mediated by ASK1 in which neuronal death is also prominent, including amyotrophic lateral sclerosis, Alzheimer’s disease^34^ and Parkinson’s disease^35^. In addition, we observed that the C-terminal TRAF domain, with which ASK1 interacts directly, is vital for ASK1 phosphorylation and the subsequent suppression of the anti-apoptotic Akt signalling pathway. Several studies have demonstrated that TRAF1 mediates proapoptotic signals after its cleavage into two fragments by caspase-8, which is consistent with our findings^16^,^36^,^37^. In addition, overexpression of the C-terminal but not the N-terminal cleavage product increases cell death. Nevertheless, our data conflict with a report that ASK1 is activated by the overexpression of TRAF2, TRAF5 and TRAF6 but not by TRAF1 overexpression in HEK293 cells^38^, although this conclusion is based on the observation that TRAF1 overexpression did not activate the MKK6/p38 pathway downstream of ASK1. However, we demonstrated that, at least during ischaemic neuronal injury, TRAF1 activates only the ASK1/JNK signalling pathway and not the p38 MAPK pathway.

Inhibition of prominent mediators of neuronal death has been associated with greater neuroprotective efficacy than targeting the downstream mitochondrial pathway^1^. In our study, we depict TRAF1 as a critical pro-death modulator that acts on the upstream MKK/JNK signalling pathways. Interestingly, Repici *et al*.^19^ reported that the activation of MKK4, JNK and c-Jun was only marginally triggered at 3 h post-stroke and eventually plateaued at 6 h post-stroke. We observed that the temporal expression pattern of TRAF1 followed a similar trend, with mild but significant induction at 2 h post-injury and a greater than 6-fold upregulation at 6 h and beyond, further supporting the hypothesis that TRAF1 is required to activate the MKK/JNK/c-Jun pathway. Indeed, upon subjection to I/R for 6 h, the expression of phosphorylated MKK4/7, JNK and c-Jun was increased by 1.4–2.4-fold in TG2-TRAF1 mice and decreased by 25–51% in TRAF1-KO mice. Consistent with the protective effects of TRAF1 ablation, both genetic and pharmacological ablation of JNK results in reduced volumetric stroke lesions^7^,^39^. Previous studies have demonstrated that JNK exerts pro-apoptotic effects via induction of Fas and Bax, together with the release of cytochrome C and the activation of caspase-9 and caspase-3, but does not alter the expression levels of FasL and neuroprotective Bcl2, which was observed in our model^20^. Therefore, in addition to JNK, TRAF1 may function through an alternative mechanism. Recent studies have suggested that the activation of apoptotic cascades during stroke likely results from dysfunction of the Akt pro-survival pathway^40^. However, whether the TRAF1/ASK1 pathway interferes with Akt-mediated neuronal survival during stroke remains to be determined. In this study, we showed that the TRAF1/ASK1 cascade negatively regulates the phosphorylation of Akt and its downstream effectors. In addition, ASK1 inactivation by genetic knockdown or RNA interference augments the hypertonicity-induced Akt phosphorylation and activation^23^, whereas the TNF-α-induced ASK1 phosphorylation decreases Akt activation^41^. In summary, we have demonstrated that both JNK and Akt are targets of the TRAF1/ASK1 pathway. Orchestration of the two counteractive effects may well explain the marked attenuation of infarct progression observed upon inhibition of a single mediator, TRAF1. Intriguingly, we observed that the Fas/FasL pathway is indispensable for TRAF1-mediated neuronal death. In alignment with our findings, Le Niculescu reported that JNK is required for Fas ligand induction in neuronal cells^26^. Thus, our data further strengthen the previous findings that Fas/FasL is critical for neuronal apoptosis in stroke.

Our data support a pro-apoptotic role for TRAF1 within a period of hours to days post-stroke. In our experiments, TRAF1 activity was mediated by its ability to directly interact with and activate ASK1, and inhibition of the TRAF1/ASK1 signalling pathway not only rescued neurons but also restored neurological function. Consequently, we are cautiously optimistic that TRAF1-mediated activation of the ASK1 pro-death signalling pathway is a promising therapeutic target for stroke management, likely beyond the accessible window of opportunity for tPA therapy.

## Methods

### Animals

All animal procedures were approved by the Animal Care and Use Committee of Renmin Hospital of Wuhan University. Animal experiments were performed in accordance with the National Institutes of Health Guide for the Care and Use of Laboratory Animals (NIH Publication No. 80-23 revised in 1996). C57BL/6 TRAF1-KO mice were purchased from Jackson Laboratory (Bar Harbor, ME, USA; Stock No. 008076), and the mice were genotyped using PCR analysis with the primers 5′-GCCAGAGGCCACTTGTGTAG-3′, 5′-CAGAACCCCTTGCCTAATCC-3′ and 5′-TCCTAGAGGCCTGCTGCTAA-3′. To generate TRAF1 transgenic mice, we cloned full-length murine TRAF1 cDNA downstream of the neuron-specific promoter platelet-derived growth factor as a 1229-bp XhoI-BamHI fragment using amplification with the primers 5′-GAACTCGAGCCACCATGGCCTCCAGCTCAGCCCC-3′ (forward) and 5′-GAAGGATCCTAAGCACTAGTGTCCACAA-3′ (reverse). This construct drives the preferential expression of TRAF1 in neurons. Transgenic mice were produced by microinjecting the construct into fertilized embryos (C57BL/6 background), and four independent transgenic lines were established. PCR analysis of tail genomic DNA was performed using the primers 5′-AAGGGTGGCAACTTCTCCTC-3′ (forward) and 5′-ATAAGGAATGGACAGCAGGG-3′ (reverse) to genotype the mice. Only 11–12-week-old (25–30 g) males were used. All mice were housed in an environment with controlled light (12 h light/dark), temperature and humidity, with food and water available *ad libitum*.

### Mouse cerebral I/R model

Focal cerebral ischaemia was induced by occluding the left MCA using the intraluminal filament technique^42^,^43^,^44^. Briefly, the mice were anesthetized with 2.5–3% isoflurane in O_2_. The rectal temperature was maintained at 37±0.5 °C using a homoeothermic blanket. rCBF was measured using a laser-Doppler flowmetry instrument (Periflux System 5010; Perimed, Järfälla, Sweden) with a flexible probe affixed to the skull (2 mm posterior and 5 mm lateral to the bregma). To achieve tMCAO, a 6-0 silicon-coated monofilament surgical suture (Doccol, Redland, CA, USA) was inserted into the left external carotid artery, advanced into the internal carotid artery and wedged into the cerebral arterial circle to obstruct the origin of the MCA. A decline in rCBF >75% confirmed the interruption of blood flow in the MCA. Sixty minutes after MCAO induction, the filament was withdrawn, and a return to >70% of basal cerebral blood flow within 10 min of suture withdrawal confirmed reperfusion of the MCA territory. The mice underwent reperfusion for the durations indicated in the text. The mice in which the filament was withdrawn immediately after the decline in rCBF were used as sham controls. Heart rate, systolic blood pressure and diastolic blood pressure were monitored in randomly selected, conscious mice using the non-invasive tail-cuff method. We measured body weight and arterial blood gases before surgery.

The experimental groups were WT (*n*=74), TRAF1 KO (*n*=70), NTG (*n*=56) and TG-TRAF1 (*n*=62). Analyses were conducted at the times after reperfusion indicated in the text. In all experiments, the examiners were blinded to the mouse genotypes.

### India ink staining

The integrity of the cerebral vasculature of the experimental mice was confirmed by India ink staining^42^,^43^,^44^. Briefly, the animals were anesthetized by intraperitoneal injection of 50 mg kg^−1^ sodium pentobarbital (Sigma, St Louis, MO, USA) in 10 ml physiological saline and perfused through the left cardiac ventricle with 2 ml preheated staining solution containing 10% (W/V) gelatin (Amresco, Solon, OH, USA) and 50% (V/V) India ink (Solarbio, Beijing, China). Perfusion was stopped when the tissues, including the tongue, lips and gums, turned black. The isolated brains were immersed in 10% buffered formalin for 24 h before examination. Vessels of the circle of Willis and their branches were photographed using a Nikon D700 digital camera.

### Neurological deficit scores

Neurological deficits were assessed after 24 and 72 h of reperfusion using a nine-point scale as follows: absence of neurological deficit, 0; left forelimb flexion upon suspension by the tail or failure to fully extend the right forepaw, 1; left shoulder adduction upon suspension by the tail, 2; reduced resistance to a lateral push towards the left, 3; spontaneous movement in all directions with circling to the left only if pulled by the tail, 4; circling or walking spontaneously only to the left, 5; walking only when stimulated, 6; no response to stimulation, 7; and stroke-related death, 8 (ref. 45).

### Measurement of infarct volume

Following reperfusion, the mice were euthanized, and their brains were immediately removed. Mouse brains were cut into 1-mm coronal sections and a total of seven sections were prepared^46^. The sections were incubated with TTC staining solution for 15 min at 37 °C before fixed in 10% formalin solution overnight. The sections were imaged, and the volume of the infarct area was quantified with Image-Pro Plus 6.0. To exclude the effects of brain oedema, the percentage of infarct volume was calculated as follows: (volume of the contralateral hemisphere minus the volume of the non-lesioned ipsilateral hemisphere)/(contralateral volume times 2). We also calculated the relative oedema volume (%) as follows: (volume of the contralateral hemisphere minus the volume of the ipsilateral hemisphere)/contralateral volume. Finally, we calculated the infarct and oedema volumes by integrating their areas over seven brain sections.

### Immunofluorescence and TUNEL staining

The animals were anesthetized with sodium pentobarbital and perfused with 0.1 mol l^−1^ sodium phosphate buffer (pH 7.4) under 100 mm Hg pressure for 5 min and with 4% paraformaldehyde in phosphate buffer for 15 min. Immunocytochemistry was performed as previously described^42^. Briefly, the brains were rapidly removed and fixed in the same fixative solution for 6–8 h at room temperature, followed by immersion overnight at 4 °C in phosphate buffer containing 30% sucrose. The brains were embedded in OCT solution, and cryosections were prepared. The neurons were cultured on cover slips and fixed in ice-cold acetone. Next, the sections on cover slips were washed in PBS containing 10% goat serum and incubated overnight with the following primary antibodies at 4 °C: mouse anti-NeuN (LV1825845; 1:200; Millipore, Temecula, CA, USA), chicken anti-MAP2 (Ab5392; 1:100; Abcam, Cambridge, UK), rabbit anti-cleaved caspase-3 (no. 19662; 1:100; Cell Signaling Technology, Danvers, MA, USA) and anti-TRAF1 (no. ab129279, 1:100, Abcam). The sections were washed in PBS and incubated in the appropriate secondary antibody for 1 h. The secondary antibodies used were goat anti-chicken IgY (H&L, DyLight 488, ab96947, Abcam), goat anti-mouse IgG Alexa Fluor 568 conjugate (A11004; Invitrogen, Carlsbad, CA, USA) and anti-rabbit IgG Alexa Fluor 568 conjugate (A11011, Invitrogen). The nuclei were stained with 4',6-diamidino-2-phenylindole (DAPI). Images were acquired with a fluorescence microscope (OLYMPUS DX51) and DP2-BSW software (version 2.2), and the images were analysed with Image Pro Plus 6.0. For NeuN and TUNEL co-staining, the sections were first stained with NeuN antibody, followed by TUNEL staining with an ApopTag Plus In Situ Apoptosis Fluorescein Detection Kit (S7111, Millipore), according to the manufacturer’s protocol.

### Tissue preparation

For quantitative real-time PCR and western blot analysis, the animals were anesthetized with 3% sodium pentobarbital and perfused via the left ventricle with cold sodium phosphate; the brains were then immediately removed. To collect the tissue in an unbiased manner that reflected the global extent of the infarcts, the olfactory bulbs and 1-mm sections of the anterior and posterior brain tissue were excised. We then collected the remaining tissues of the ipsilateral (including the infarct and peri-infarct areas) and contralateral (normal) hemispheres. All tissues were immediately frozen in liquid nitrogen and stored at −80 °C.

### Quantitative real-time PCR

Total RNA was isolated from frozen tissue using TRIZOL reagent (Invitrogen) following the manufacturer’s protocol, and 2 μg of RNA was reverse-transcribed with the Transcriptor First Strand cDNA Synthesis Kit (Roche, Indianapolis, IN, USA). Quantitative RT-PCR analysis was performed using the LightCycler 480 SYBR Green 1 Master Mix (Roche) and the LightCycler 480 QPCR System (Roche). The PCR conditions were 95 °C for 10 min; 40 cycles of 95 °C for 10 s, 60 °C for 10 s and 72 °C for 20 s; and a final extension at 72 °C for 10 min. GAPDH served as the internal control. The following primer pairs were used:

Bax forward, 5′-TGAGCGAGTGTCTCCGGCGAAT-3′;

Bax reverse, 5′-GCACTTTAGTGCACAGGGCCTTG-3′;

Bcl2 forward, 5′-TGGTGGACAACATCGCCCTGTG-3′;

Bcl2 reverse, 5′-GGTCGCATGCTGGGGCCATATA-3′;

Fas forward, 5′-AATGGGGGTACACCAACCTGCG-3′;

Fas reverse, 5′-TTCACACGAGGCGCAGCGAA-3′;

FasL forward, 5′-GGGTGCCATGCAGCAGCCCATGAAT-3′;

FasL reverse, 5′-TGGCGGCAGTGGGAGTGGTTGTGAT-3′;

GAPDH forward: 5′-ACTCCACTCACGGCAAATTC-3′; and

GAPDH reverse: 5′-TCTCCATGGTGGTGAAGACA-3′.

### Western blot analysis

Proteins were extracted from brains or cultured cells and homogenized in lysis buffer. Proteins (50 μg) were separated on 8–12% SDS–PAGE gels and transferred to PVDF (polyvinylidene difluoride) membranes (Millipore, Bedford, MA). The membranes were blocked in TBST (Tris-buffered saline Tween-20) containing 5% skim milk powder for 1 h at room temperature and incubated with primary antibodies overnight at 4 °C. Next, the membranes were incubated with secondary antibodies (peroxidase-affinipure goat anti-mouse IgG (H+L) (115-035-003), or peroxidase-affinipure goat anti-rabbit IgG (H+L) (111-035-003); Jackson ImmunoResearch Laboratories, Lincoln, NE, USA) for 1 h at room temperature. Membranes were treated with ECL reagents (170–5061; Bio-Rad) before visualization using FlourChem E imager (Proteinsimple, FlourChem E) according to the manufacturer’s instructions. The following primary antibodies were used: rabbit anti-caspase-3 (no. 9662), anti-caspase-9 (no. 9504), anti-cleaved caspase-9 (Asp353; no. 9509), anti-Akt (no. 4691) and anti-phospho-Akt (Ser473; no. 4060), anti-mTOR (no. 2983), anti-phospho-mTOR (Ser2448; no. 2971), anti-MKK4/7 (no. 9264), anti-phospho-MKK4/7 (Ser189/207; no. 9231), anti-phospho-JNK (Thr183/185; no. 4060), anti-CREB (no. 9197), anti-phospho-CREB (Ser133; no. 9198), anti-Bax (no. 2772), anti-Bcl-2 (no. 2870) and anti-phospho-ASK1 (Thr845; no. 3765) (all from Cell Signaling Technology, Beverly, MA, USA; 1:1,000); rabbit anti-JNK1/2/3 (T178; BS3630), anti-p-c-Jun (S63; BS4045) and anti-p-c-Jun (S73; BS4046) (all from Bioworld Technology, Minneapolis, MN, USA; 1:500–1:1,000); rabbit anti-BDNF (ab72439; 1:1,000 dilution), anti-GAP43 (ab12274; 1:500–1:2,000) and anti-NGF (ab6199; 1:1,000 dilution) (all from Abcam); mouse anti-cleaved caspase-3 (Asp175; AB3623; 1:100-1:200; Millipore); and goat anti-ASK1 (N19; sc6368; 1:200) and rabbit anti-TRAF1 (H125; sc1830; 1:200) (both from Santa Cruz Biotechnology, Santa Cruz, CA, USA). Mouse anti-GAPDH (MB001; 1:5,000-1:10,000) (Bioworld Technology) served as the internal control. Full gel scans are shown in Supplementary Fig. S14.

### *In vitro* model of ischaemia/reperfusion

The cortical neurons were collected from Sprague–Dawley rats within 1 day of birth. Briefly, the cortices were dissociated by incubation for 15 min at 37 °C in 2 ml 0.125% trypsin (GIBCO, Grand Island, NY, USA), followed by the addition of 4 ml Dulbecco’s modified Eagle’s medium (DMEM)/F-12 (GIBCO) containing 20% fetal bovine serum (FBS, GIBCO) to inactivate the trypsin. The cell suspension was centrifuged for 10 min at 1,000 rpm and resuspended in DMEM containing 20% FBS. The cells were then passed through 200-μm sterile filters and seeded on plates coated with poly-L-lysine (10 mg ml^−1^, Sigma). The neurons were cultured in neurobasal medium (GIBCO) fortified with B27 (GIBCO) for 24 h at 37 °C and 5% CO_2_. AraC (10 μM, Sigma) was added to the medium 24 h after plating to inhibit cell proliferation, and the medium was changed every 48 h. The cells were cultured for 5 days before the experiments. To model ischaemia/reperfusion conditions *in vitro*, the neuronal cultures were exposed to transient OGD for 60 min and then returned to normal culture conditions for various periods. For OGD, the neurobasal medium was replaced with serum-free, glucose-free Locke’s buffer (154 mM NaCl, 5.6 mM KCl, 2.3 mM CaCl_2_, 1 mM MgCl_2_, 3.6 mM NaHCO_3_, 5 mM HEPES and 5 mg ml^−1^ gentamicin, pH 7.2), and the cultures were incubated in an experimental hypoxia chamber in a saturated atmosphere of 95% N_2_ and 5% CO_2_. Control cells cultured in the presence of normal levels of glucose were incubated for the same periods in a humidified atmosphere of 95% air and 5% CO_2_. The neurons were incubated with anti-FasL antibody (20 μg ml^−1^, Santa Cruz Biotechnology), Fas–Fc (10 μg ml^−1^, R&D Systems Inc.), Z-VAD.FMK (100 μM, V116, Sigma) or Necrostatin-1 (25 μM; N9037; Sigma) before OGD exposure as indicated.

### Construction of adenoviral vectors

Adenoviruses harbouring sequences encoding mouse TRAF1, short hairpin RNA targeting TRAF1 (shTRAF1), TRAF1 with a mutant TRAF domain (Ad-mutant), human ASK1 or dominant negative ASK1-K709R (dn-ASK1) were generated. The ORF clone of mouse TRAF1 subcloned into a pCMV6-AC-GFP shuttle vector was purchased from OriGene (MG206422). The coding sequence of human ASK1 was reverse-transcribed using the primers 5′-CCAGATTACGCTGATAGCACGGAGGCGGACGAGGG-3′ (forward) and 5′-GAAGATCTTGATTTAAGTCTGTTTGTTTCGAAAGT-3′ (reverse). dn-ASK1 was a gift from Prof. Hidenori Ichijo (The University of Tokyo, Tokyo, Japan)^25^. Recombinant adenoviruses were generated using an AdEasy vector kit (Stratagene, La Jolla, CA, USA). Inserts were cloned into the pShuttle-CMV vector. Plasmids were recombined with the pAdEasy backbone vector according to the manufacturer’s instructions and transfected into HEK293 cells using FuGENE transfection reagent (E2312, Roche). Recombinant adenoviruses were plaque-purified, titered to 10^9^ PFU ml^−1^, and verified by restriction digestion. Ad-mutant recombinant adenovirus harbouring TRAF1 with a mutant TRAF domain was generated using a similar protocol. To generate shTRAF1, the hairpin-forming oligonucleotides TRAF1-f, 5′-GATCCGCGTGTGTTTGAGAACATTGTTCTCGAGAACAATGTTCTCAAACACACGTTTTTTACGCGTG-3′ and TRAF1-r, 5′-AATTCACGCGTAAAAAACGTGTGTTTGAGAACATTGTTCTCGAGAACAATGTTCTCAAACACACGG-3′ were synthesized, annealed and subcloned into the shuttle vector distal to the U6 promoter. Using a similar process, recombinant adenovirus was generated. Cultured cortical neurons were transfected with adenovirus at an MOI of 100 for 48 h.

### Plasmid construct and transfection

To construct the EGFP-myc-TRAF1 plasmid, the mouse TRAF1 insert was amplified using PCR with the primers TRAF1-5′ and TRAF1-3′, digested with BamHI and XhoI, and ligated into pEGFP-myc-C1, which was digested with BamHI and XhoI. To generate TRAF1 fragments consisting of residues 1–261 and 261–409, EGFP-myc-TRAF1 was PCR amplified using the primer pairs TRAF1-5′ and TRAF1-N-3′ and TRAF1-C-5′ and TRAF1-3′, respectively. The PCR products were digested with BamHI and XhoI and ligated into pEGFP-myc-C1 to create an in-frame fusion with EGFP-myc. To construct recombinant Flag-cherry-ASK1, the human ASK1 insert was amplified with ASK1-5′ and ASK1-3′, digested with SalI, and ligated into pCMV-Flag-cherry. Flag-cherry-ASK1 was PCR-amplified with the primer pairs ASK1-5′ and ASK1-N1-3′, ASK1-C1-5′ and ASK1-C1-3′, and ASK1-C2-5′ and ASK1-3′ to obtain ASK1 fragments consisting of residues 1–678, 678–936 and 936–1375, respectively, and the PCR products were digested with BamHI and SalI, BamHI and SalI, or BglII and SalI, respectively. These products were ligated into pCMV-Flag-cherry digested with BamHI and XhoI to create an in-frame fusion with Flag-cherry. All plasmids were verified through sequencing. Plasmid DNA was transfected into cells at 50% confluence in 12-well plates (Greiner Bio One, Monroe, NC, USA) with FuGENE transfection reagent (E2312, Roche) for 48 h, according to the manufacturer’s instructions.

### Immunoprecipitation

Cultured HEK293T cells were co-transfected with pEGFP-myc-TRAF1 and pFlag-cherry-ASK1 for 48 h with FuGENE transfection reagent (Roche). Primary cortical neurons were subjected to OGD exposure with or without Ad-TRAF1 infection, as indicated. The cells were lysed in IP buffer (20 mM Tris-HCl, pH 8.0, 100 mM NaCl, 1 mM EDTA, and 0.5% NP-40) containing protease inhibitor cocktail (Roche). After 20 min of incubation at 4 °C with gentle vortex mixing, the cell lysates were centrifuged at 13,000 × *g* for 10 min at 4 °C. Sample aliquots (500 μl) were precleared with 10 μl Protein A/G-agarose beads (11719394001, 11719386001, Roche) and incubated with 1 μg of antibody or control IgG overnight at 4 °C, according to the manufacturer’s recommendations. The beads were washed 5–6 times with 800 μl cold IP buffer, resuspended in 1 × loading buffer, and heated at 95 °C for 5 min. The cell lysates and immunoprecipitates were analysed through western blotting, as described above.

### GST pull-down assay

GST-TRAF1 was constructed using pGEX-4T-1 and expressed in *E. coli* Rosetta (DE3) cells. The cultures were induced with IPTG; 10 ml aliquots of cells were harvested, and fusion proteins were purified using glutathione-Sepharose 4B beads (GE Healthcare, Chalfont St Giles, UK), according to the manufacturer’s instructions. The lysates from Flag-cherry-ASK1-transfected HEK293T cells were added to the beads and incubated for 4 h at 4 °C in IP buffer supplemented with protease inhibitor cocktail. The GST tag served as a negative control. The beads were extensively washed with IP buffer and analysed by western blotting.

### Confocal microscopy

HEK293T cells were seeded on coverslips in 24-well plates. After co-transfection of pFlag-cherry-ASK1 and pEGFP-myc-TRAF1 for 48 h, the cells were fixed in 4% fresh paraformaldehyde for 15 min, permeabilized with 0.2% Triton X-100 in PBS for 5 min and incubated in Image-IT FX signal enhancer (I36933, Invitrogen) for 30 min. The cells were washed three times with TBST and stained with 1 g ml^−1^ DAPI for 15 min. The slides were mounted with mounting solution (D2522, Sigma), and images were obtained with a confocal laser-scanning microscope (Fluoview 1000, Olympus).

### Analysis of cell viability and LDH release

Cell viability was evaluated using a non-radioactive cell counting kit-8 (CCK-8) assay (CK04, Dojindo, Kumamoto, Japan), according to the manufacturer’s instructions. LDH release was analysed using a colorimetric LDH cytotoxicity assay (G1782, Promega, Madison, WI). Three independent experiments were performed.

### Statistical analysis

Data are expressed as the mean±standard deviation (s.d.). Differences among groups were determined using analysis of variance, followed by a post hoc Tukey’s test. Comparisons between two groups were performed using an unpaired Student’s *t*-test. All *in vivo* and imaging studies were performed in a blinded manner. *P*<0.05 was considered statistically significant.

## Author contributions

Y.-Y.L. designed and conducted *in vivo* experiments and analysed data, Z.-Z.L. analysed data, drafted the manuscript and was involved in *in vitro* study design; D.-S.J. performed western blot and analysed data; L.W. contributed to data analysis; Y.Z. performed immunofluorescence; K.C., X.-F.Z., Y.L. and X.-D.Z. conducted *in vitro* experiments and contributed to data analysis; G.-C.F., Y.C., Q.Y., Y.Z. and X.-D.Z. contributed to the analysis of the data, the design of the study and the writing of the manuscript; H.L. and D.-P.L. designed the entire experiments, supervised and funded the study, and contributed to data analysis and to the writing of the manuscript; and H.L. and D.-P.L. contributed equally to the work.

## Additional information

**How to cite this article:** Lu, Y.-Y. *et al*. TRAF1 is a critical regulator of cerebral ischaemia–reperfusion injury and neuronal death. *Nat. Commun.* 4:2852 doi: 10.1038/ncomms3852 (2013).

## Supplementary Material

Supplementary Information Supplementary Figures S1-S14 and Supplementary Table S1
