# Supplementary material for: TRAF1 is a critical regulator of cerebral ischaemia–reperfusion injury and neuronal death
Source: Nat Commun. 2013 Nov 28;4:2852. doi: 10.1038/ncomms3852 (PMC3868160; doi:10.1038/ncomms3852)
Supplement: Supplementary Information — Supplementary Figures S1-S14 and Supplementary Table S1 [file ncomms3852-s1.pdf]

Supplementary Information

TRAF1 is a Critical Regulator of Cerebral Ischaemia-Reperfusion Injury and Neuronal Death

Yan-Yun Lu,[1, 2*] Zuo-Zhi Li,[3*] Ding-Sheng Jiang,[1, 2*] Lang Wang,[1, 2] Yan Zhang,[1, 2] Ke Chen,[4] Xiao-Fei Zhang,[4] Yi Liu,[4] Guo-Chang Fan,[5] Yingjie Chen,[6] Qinglin Yang,[7] Yan Zhou,[4] Xiao-Dong Zhang,[4] De-Pei Liu,[3] Hongliang Li,[1, 2]

1, Department of Cardiology, Renmin Hospital of Wuhan University, Wuhan 430060, PR China; 2, Cardiovascular Research Institute of Wuhan University, Wuhan 430060, PR China; 3, State Key Laboratory of Medical Molecular Biology, Department of Biochemistry and Molecular Biology, Institute of Basic Medical Sciences, Chinese Academy of Medical Sciences & Peking Union Medical College, Beijing 100005, China; 4, College of Life Sciences, Wuhan University, Wuhan 430072, PR China;5, Department of Pharmacology and Cell Biophysics, University of Cincinnati, Cincinnati, OH 45267-0575, USA; 6, Cardiovascular Division, University of Minnesota, Minneapolis, MN 55455, USA;7, Department of Nutrition Sciences, University of Alabama at Birmingham, Birmingham, AL 35294-3360, USA (* These authors contributed equally to the work.)

Correspondence and requests for materials should be addressed to H.L.L

(E-mail: lihl@whu.edu.cn)

Supplementary Figures S1-14

Supplementary Table S1

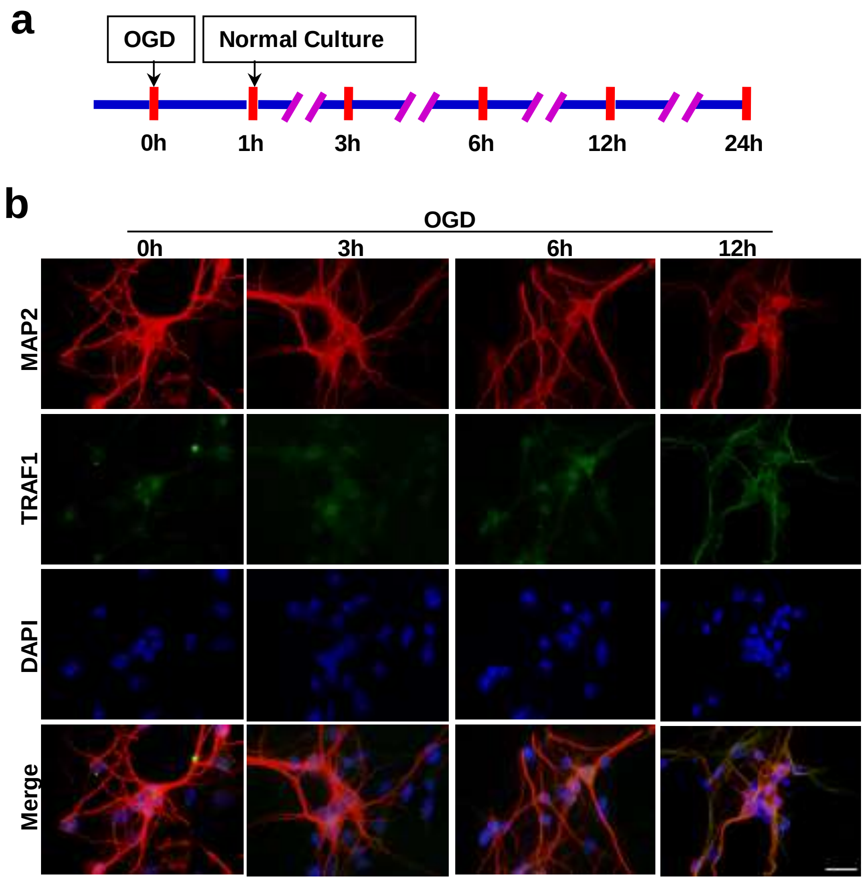

Supplementary Figure S1. OGD/reperfusion induces TRAF1 expression in neurons *in vitro*. **(a)** Time course of oxygen and glucose deprivation (OGD) of cultured cortical neurons. **(b)** Representative images of neurons co-stained for TRAF1 (green) and MAP2 (red) at the indicated times after OGD. Scale bar: 20 μm.

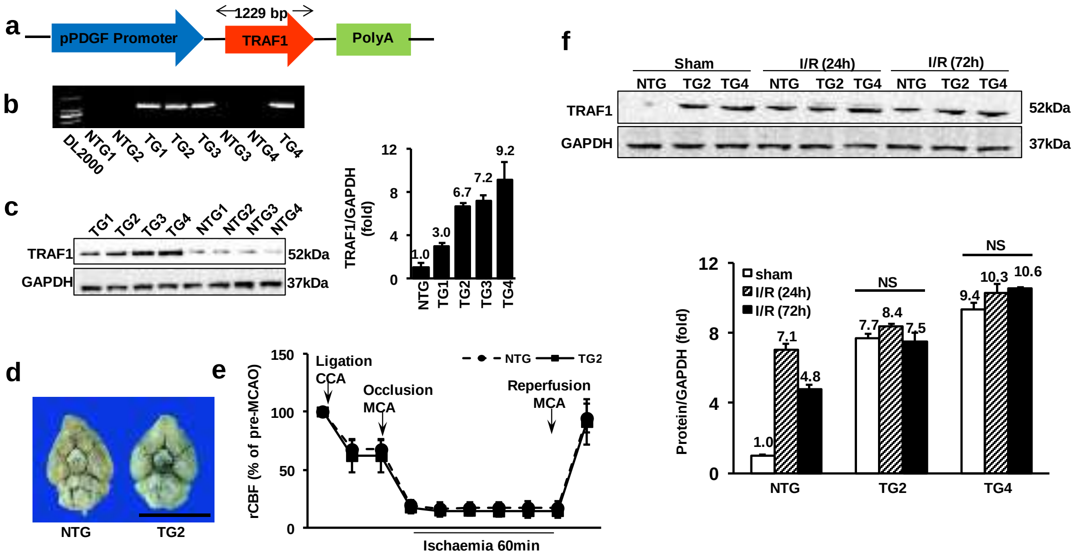

Supplementary Figure S2. Generation and characteristics of neuron-specific TRAF1 transgenic mice.

(a) Schematic representation of the transgene construct. **(b)** PCR genotyping of TG-TRAF1 and non-transgenic (NTG), wild-type (WT) mice. **(c)** Western blot analysis of TRAF1 expression in brain homogenates from four founder lines of TG1-TG4 and NTG mice (left) and relative TRAF1 levels in TG1-TG4 and NTG mice (right). **(d)** Representative images showing the integrity of the cerebral vasculature stained with India ink (n=5-8). Scale bar: 1 cm. **(e)** Regional cerebral blood flow (rCBF) in TG2-TRAF1 (TG) and NTG mice detected by Doppler before and after I/R (n=6, P>0.05 between strains, as determined by unpaired Student's *t*-test). **(f)** Immunoblots showing TRAF1 expression in TG2 and TG4 mice before and after 24 or 72 h of I/R. GAPDH served as a loading control. Bottom panel: Quantification of normalized TRAF1 levels (n=6 per time point; NS: not significant versus sham, as determined by unpaired Student's *t*-test). The data represent the mean ± s.d.

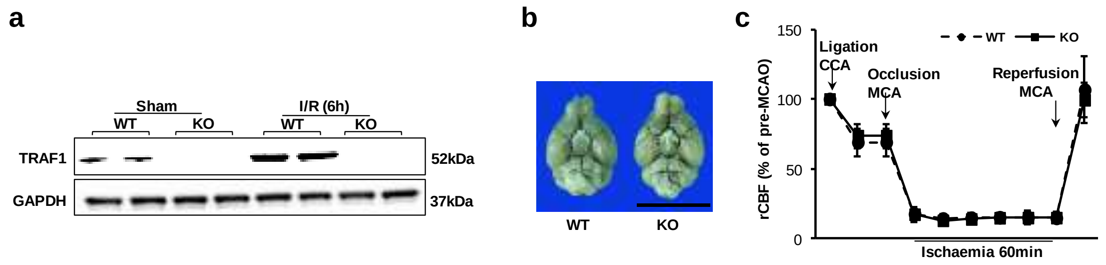

Supplementary Figure S3. Characteristics of TRAF1 knockout mice.

(**a**) Immunoblotting of TRAF1 in WT and TRAF1-KO mice. GAPDH served as a loading control. (**b**) Representative images of the integrity of the cerebral vasculature stained with India ink (n=5-8). Scale bar: 1 cm. **(c)** Regional cerebral blood flow (rCBF) detected by Doppler before and after I/R in wild-type (WT) and TRAF1-KO mice (n=6, P>0.05 between strains). The data represent the mean ± s.d. Statistical analysis was carried out by unpaired Student's *t*-test.

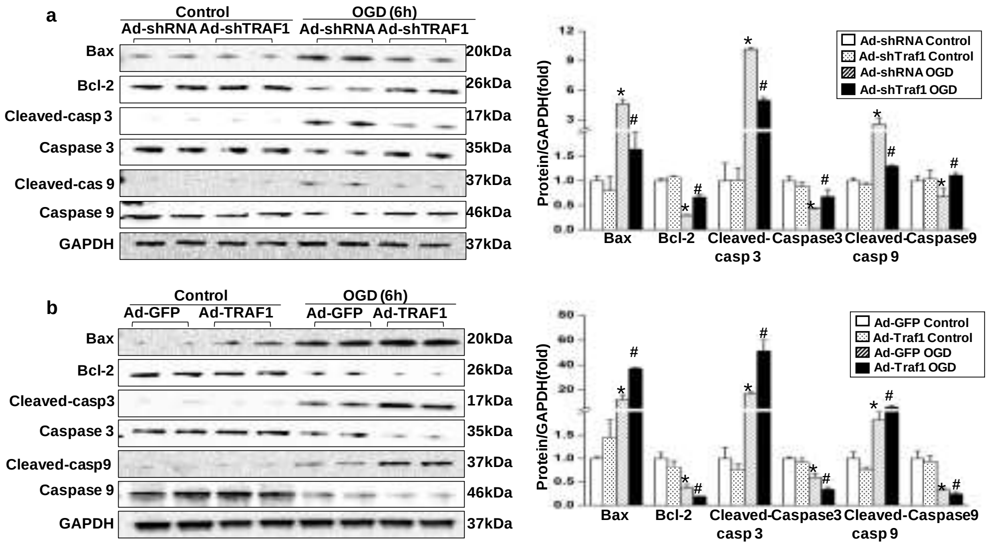

Supplementary Figure S4. TRAF1 activates OGD-induced neuronal apoptosis.

(a-b) Western (left) and densitometric (right) analysis of levels of pro-apoptotic proteins in cells infected with the indicated adenoviral vectors for 48 h and subjected to OGD for 6 h [n=6, *P<0.05 and #P<0.05 versus Ad-shRNA **(a)** and Ad-GFP (**b**) with and without OGD, respectively]. The data represent the mean ± s.d. Statistical analysis was carried out by unpaired Student's *t*-test.

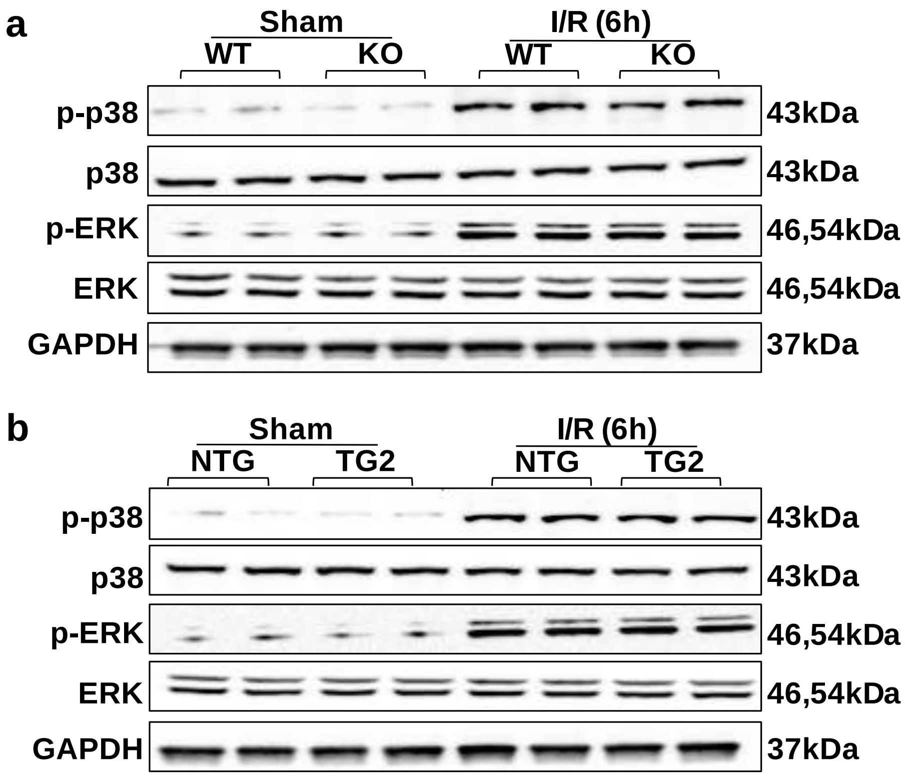

Supplementary Figure S5. TRAF1 does not affect ERK and p38 activation.

(a-b) Western analysis of levels of indicated proteins in the brains of **(a)** TRAF1-KO, wild-type (WT) and **(b)** TG2-TRAF1, non-transgenic (NTG) mice 6 h after sham surgery or ischaemia/reperfusion (I/R) (n=6), respectively.

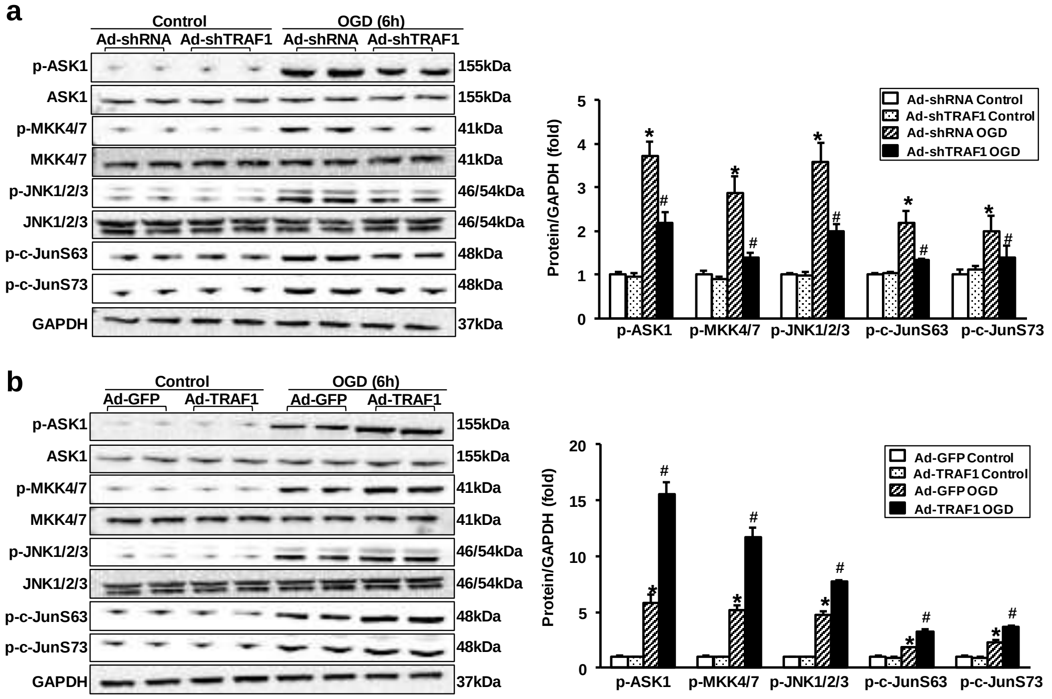

Supplementary Figure S6. TRAF1 activates ASK1/JNK *in vitro*.

(a-b) Western analysis (left) and quantification (right) of the levels of the indicated proteins in cells infected with the indicated adenoviral vectors for 48 h and subjected to OGD for 6 h (n=6, *P<0.05 and #P<0.05 versus Ad-shRNA and Ad-GFP with and without OGD, respectively). The data represent the mean ± s.d. Statistical analysis was carried out by unpaired Student's t-test.

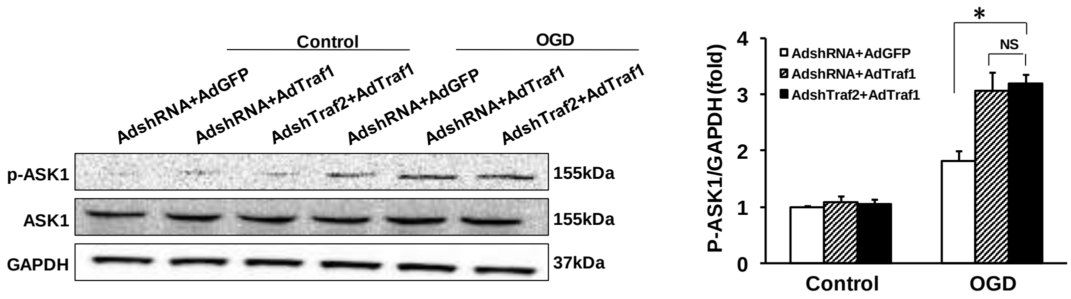

Supplementary Figure S7. TRAF2 does not affect TRAF1-ASK1 axis during I/R.

Western blot analysis of p-ASK1 and ASK1 expression in primary cortical neurons infected with indicated adenoviruses with or without OGD challenge. GAPDH served as a loading control. Right panel: quantification of normalized p-ASK1 protein levels (n=6, *P<0.05 versus the AdshRNA/AdGFP group; NS: not significant). The data represent the mean ± s.d. Statistical analysis was carried out by unpaired Student's *t*-test.

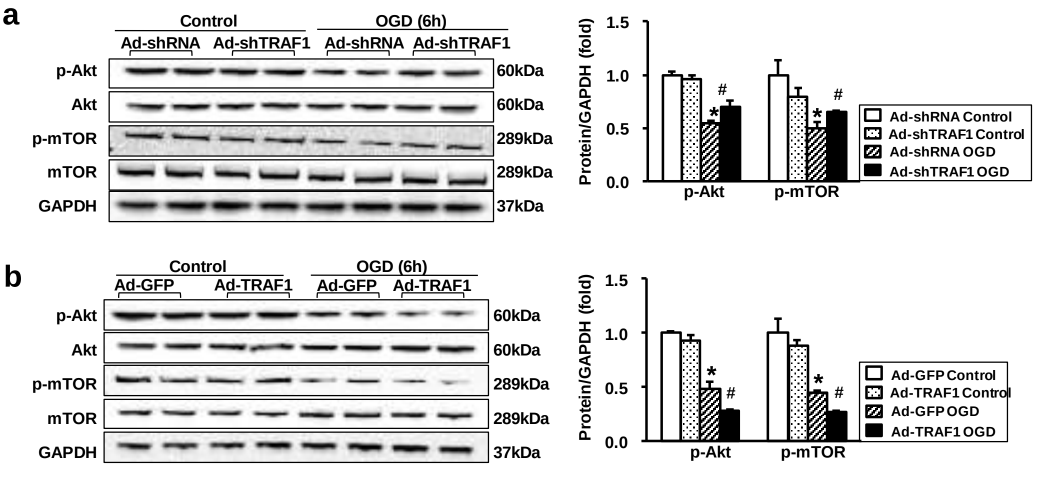

Supplementary Figure S8. TRAF1 suppresses the Akt cell survival pathway.

(a-b) Western analysis (left) and quantification (right) of the levels of the indicated proteins in cells infected with the indicated adenoviral vectors for 48 h and subjected to OGD for 6 h (n=6, *P<0.05 and #P<0.05 versus Ad-shRNA and Ad-GFP with and without OGD, respectively). The data represent the mean ± s.d. Statistical analysis was carried out by unpaired Student's t-test.

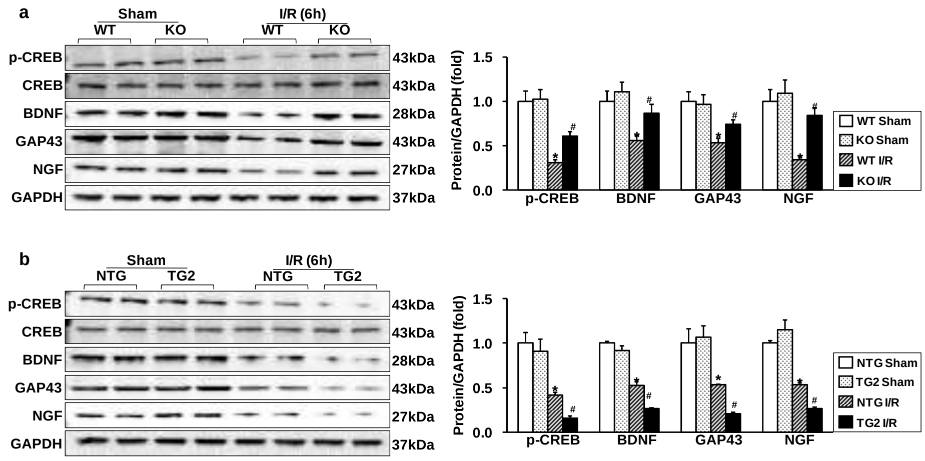

Supplementary Figure S9. TRAF1 suppresses the CREB cell survival pathway.

(a-b) Analysis of the indicated proteins in in the brains of **(a)** TRAF1-KO, wild-type (WT) and **(b)** TG2-TRAF1, non-transgenic (NTG) mice 6 h after sham surgery or ischaemia/reperfusion (I/R) (n=6, *P<0.05 versus sham-operated WT and NTG; #P<0.05 versus I/R-operated WT and NTG), respectively. GAPDH served as an internal control. The data represent the mean ± s.d. Statistical analysis was carried out by unpaired Student's t-test.

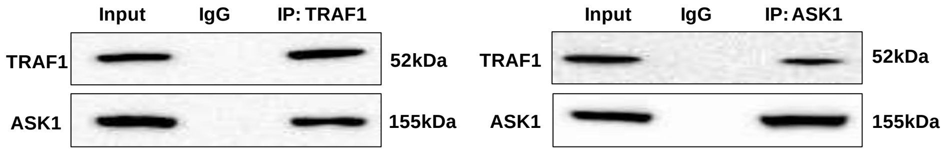

Supplementary Figure S10. Endogenous TRAF1 directly interacts with ASK1.

The lysates extracted from primary cortical neurons treated with OGD were immunoprecipited with anti-TRAF1 or anti-ASK1 and analyzed by immunoblotting using anti-TRAF1 or anti-ASK1.

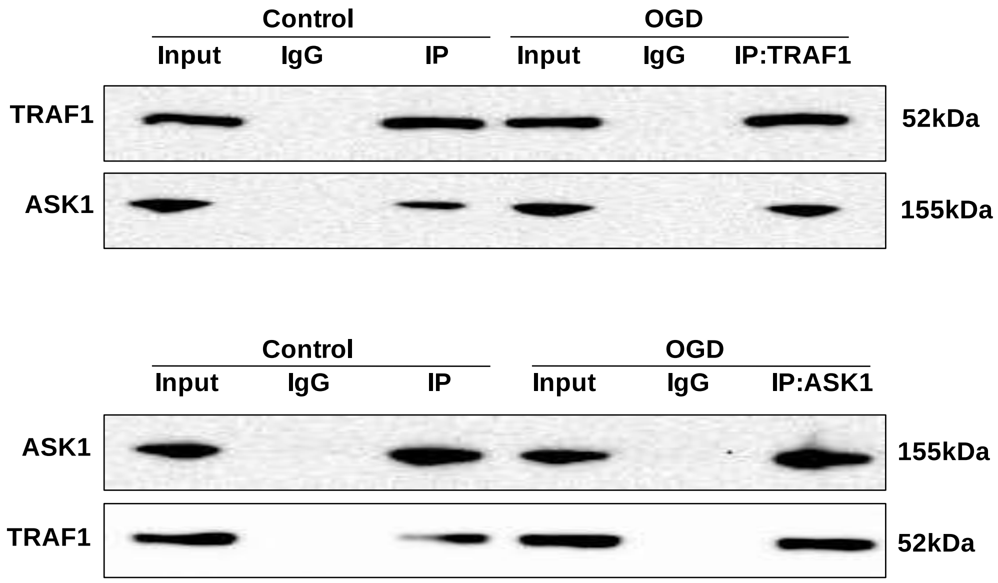

Supplementary Figure S11. I/R facilitates the interaction between TRAF1 and ASK1. Primary cortical neurons were infected with Ad-TRAF1 and cultured in normal or OGD conditions. The lysates were immunoprecipitated with anti-TRAF1 or anti-ASK1 and analyzed by immunoblotting using anti-TRAF1 or anti-ASK1 antibodies.

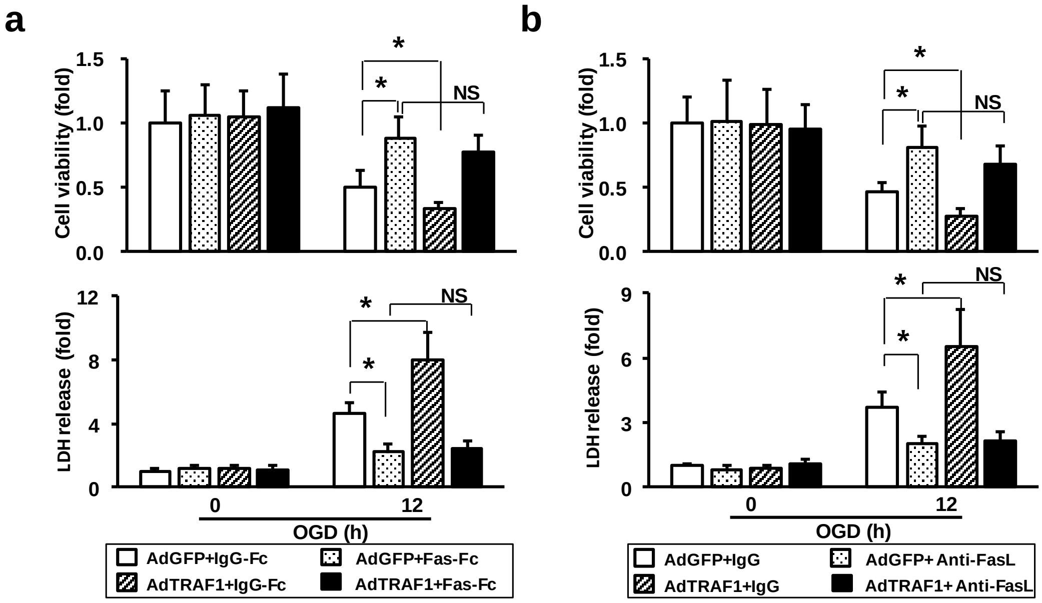

Supplementary Figure S12. The effect of Fas/FasL on TRAF1-mediated neuronal death upon ischaemic/reperfusion.

Cultured cortical neurons were infected with Ad-GFP or Ad-TRAF1 and incubated with Fas-Fc **(a)** or anti-FasL antibodies **(b)**, with or without exposure to OGD for 6 h. n=9-10, *P<0.05 versus AdGFP/IgG-Fc group. NS: not significant. The data represent the mean ± s.d. Statistical analysis was carried out by unpaired Student's t-test.

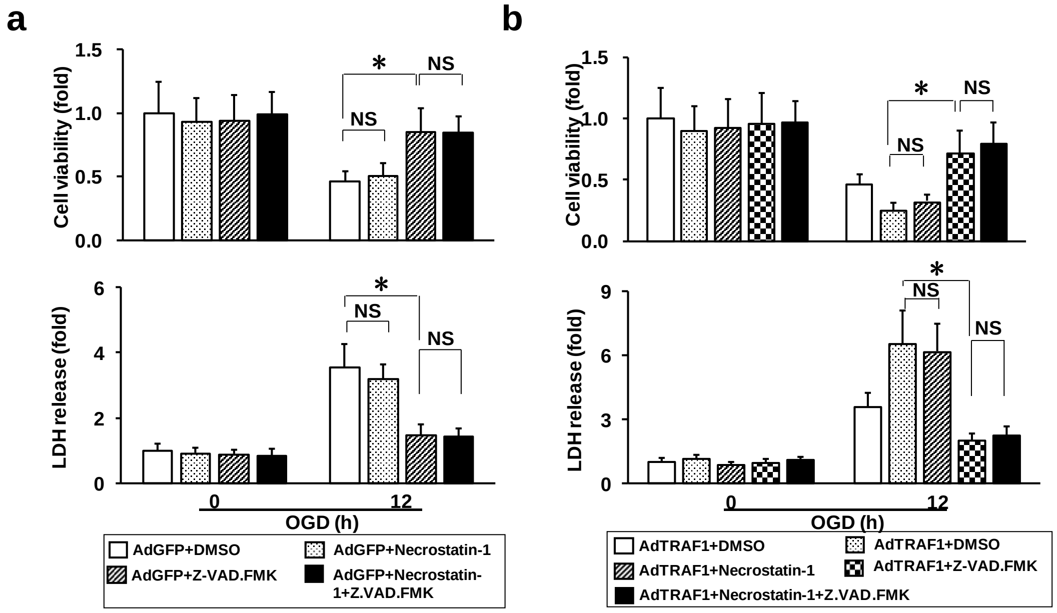

Supplementary Figure S13. TRAF1-mediated neuronal death is associated with caspase-dependent apoptosis but not necroptosis.

Primary cortical neurons were infected with AdGFP **(a)** or AdTRAF1 **(b)** and treated with Necrostatin-1 and Z-VAD.FMK, separately or jointly. Cell viability (top) and LDH release (bottom) were assayed before and after 12 h OGD/reperfusion. [n=9, *P<0.05 versus AdGFP/DMSO **(a)** or AdTRAF1/DMSO **(b)**. NS: not significant, as determined by unpaired Student's *t*-test]. The data represent the mean ± s.d.

Supplementary Figure S14. Full gel scans relating to indicated figures.

Full gels for Figure 1d

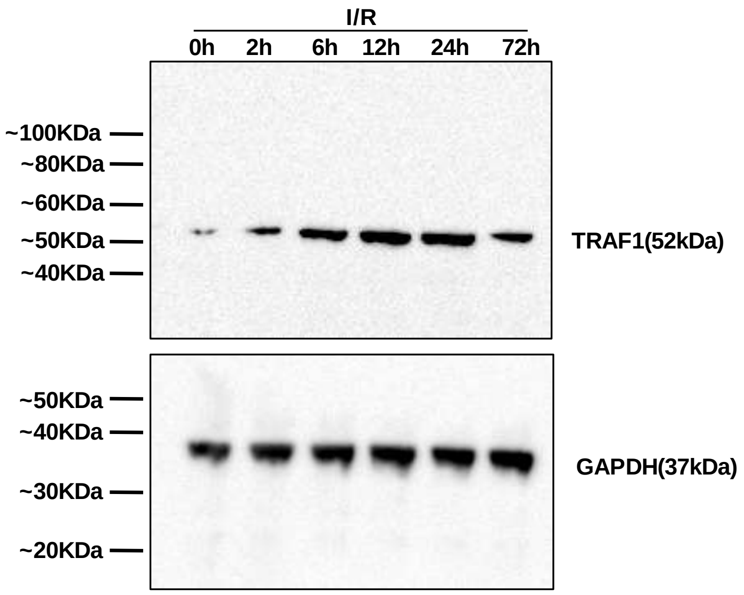

Full gels for Figure 1e

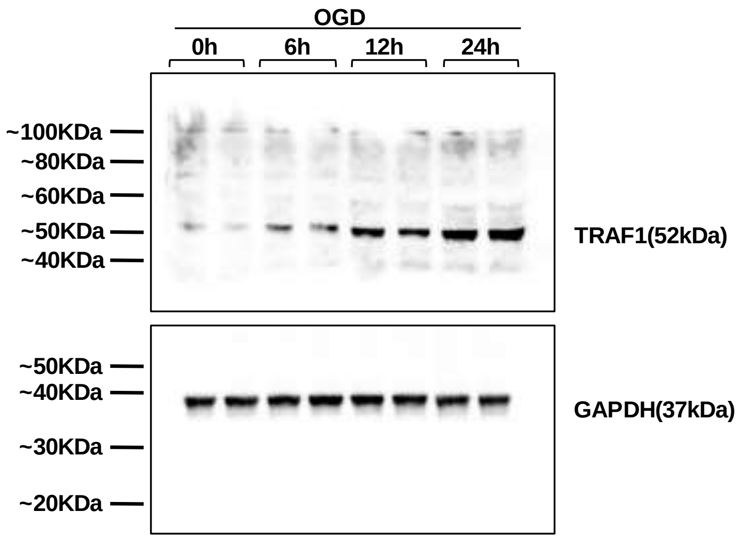

Full gels for Figure 4c

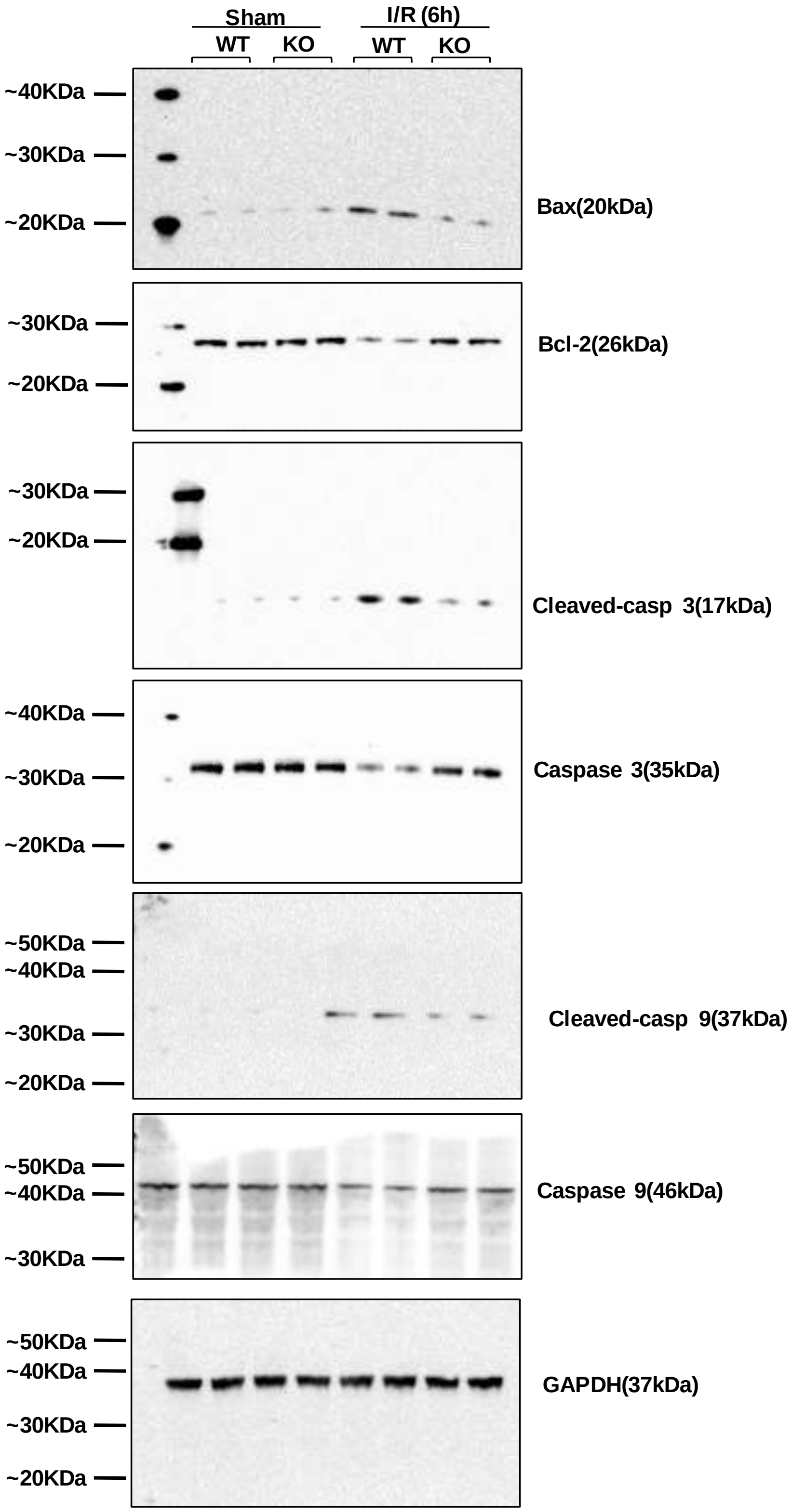

Full gels for Figure 4d

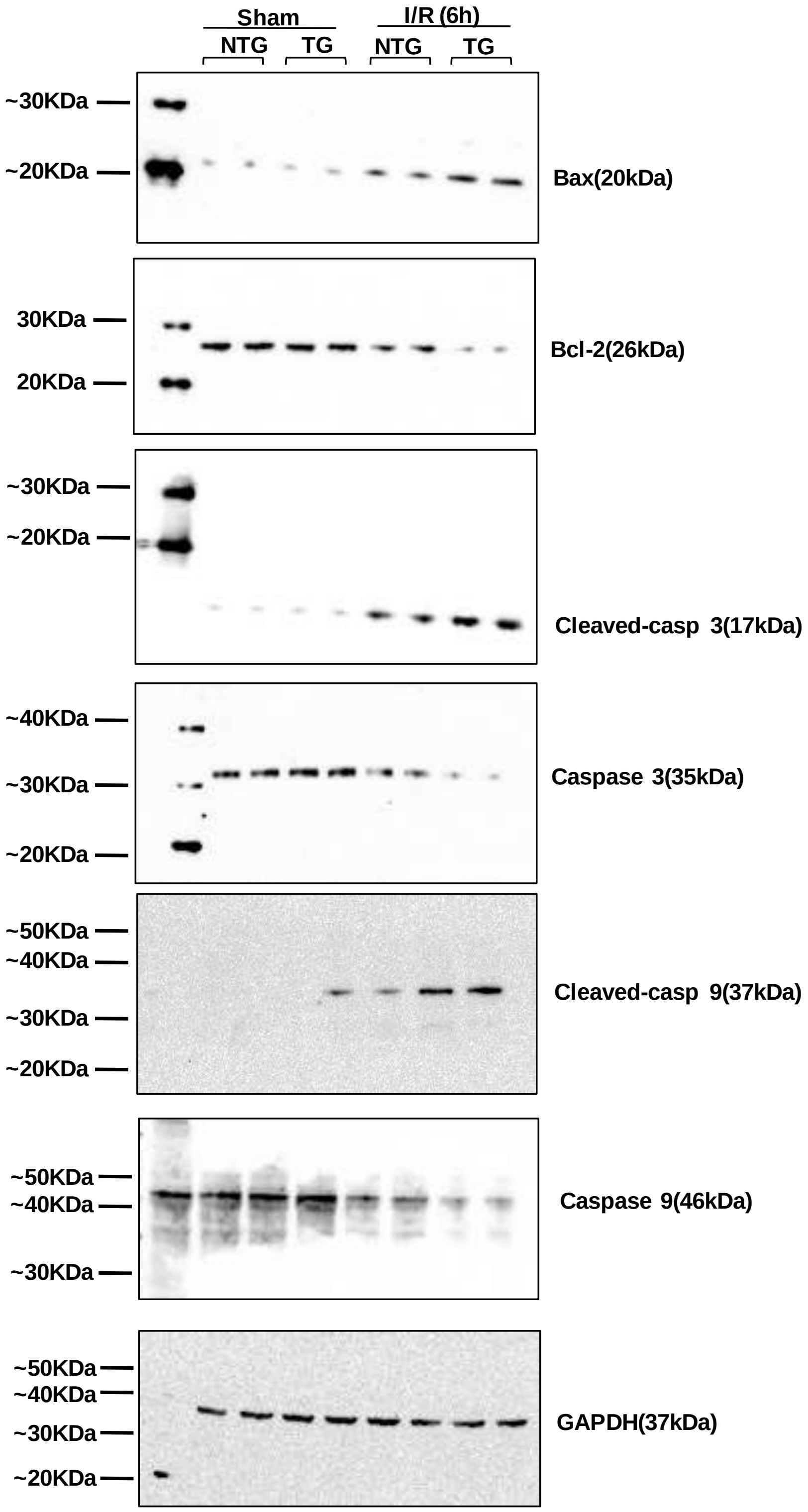

Full gels for Figure 4e

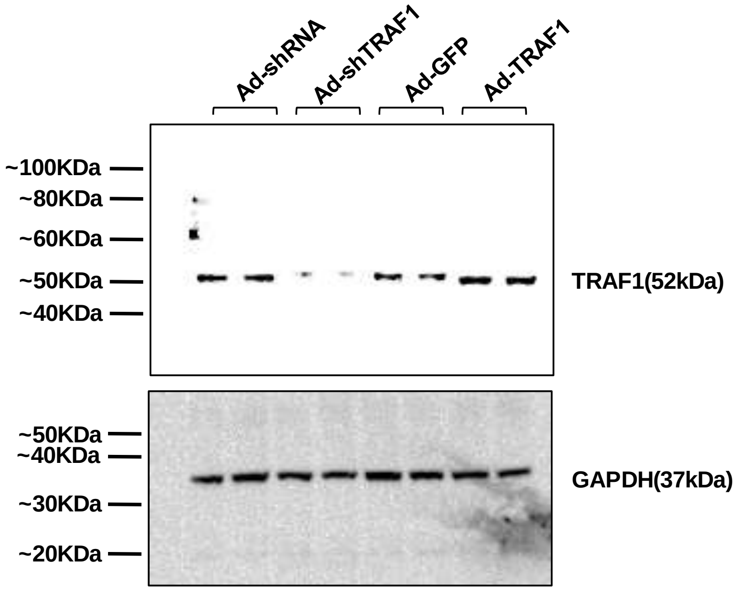

Full gels for Figure 5a

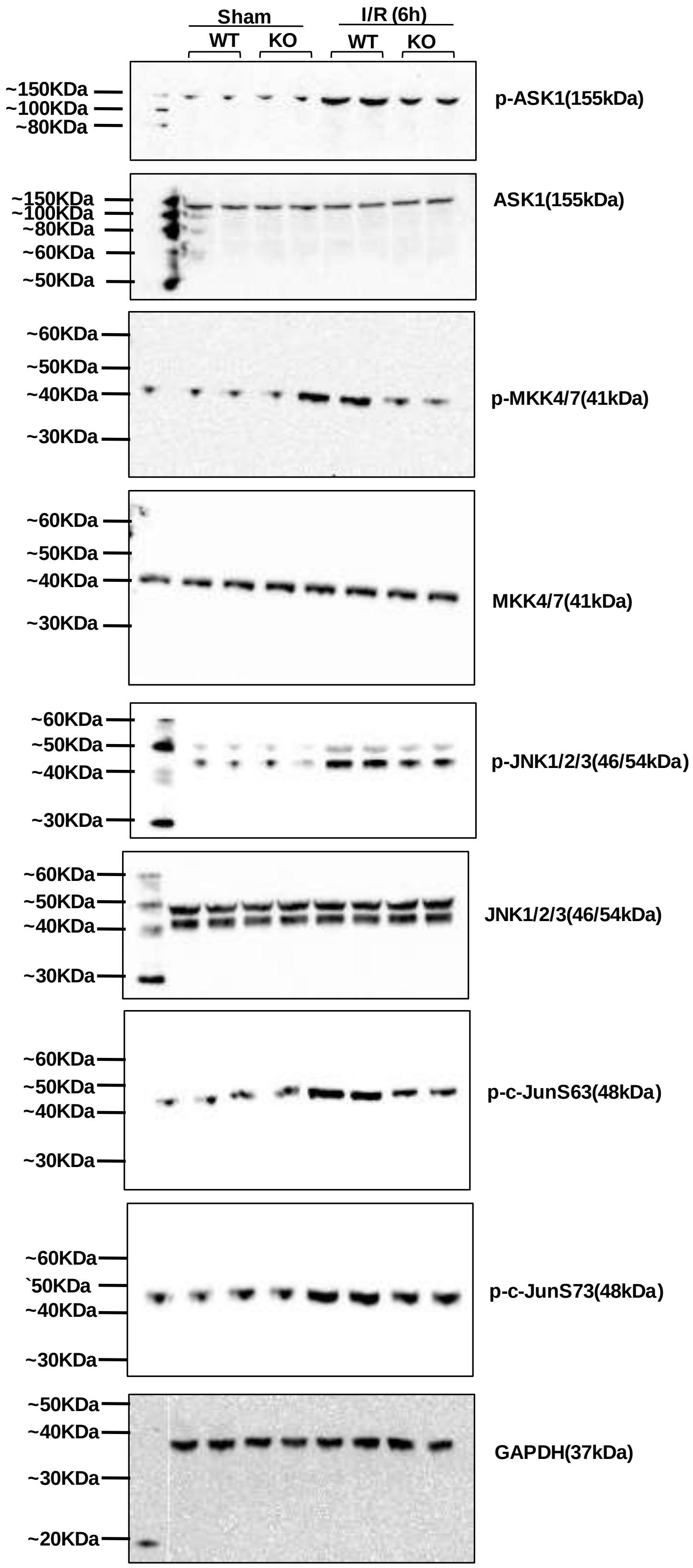

Full gels for Figure 5b

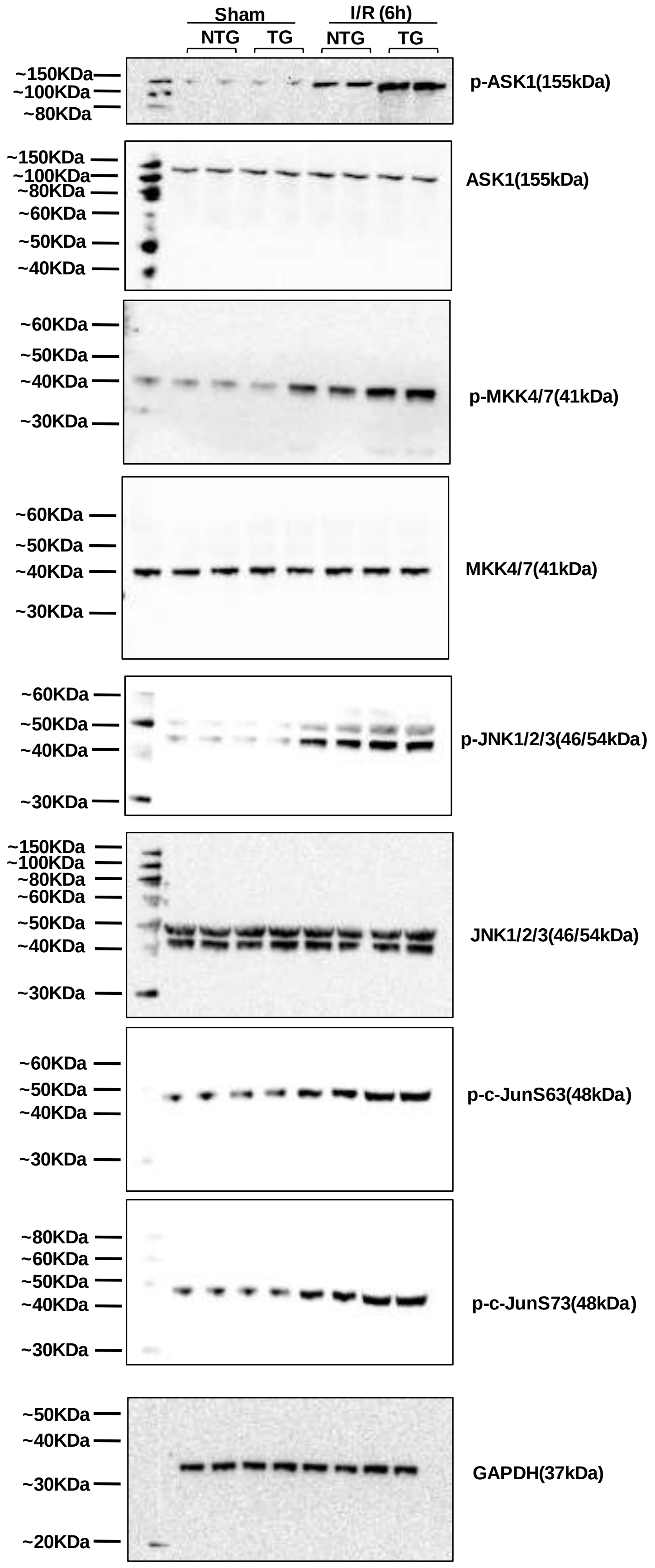

Full gels for Figure 5c

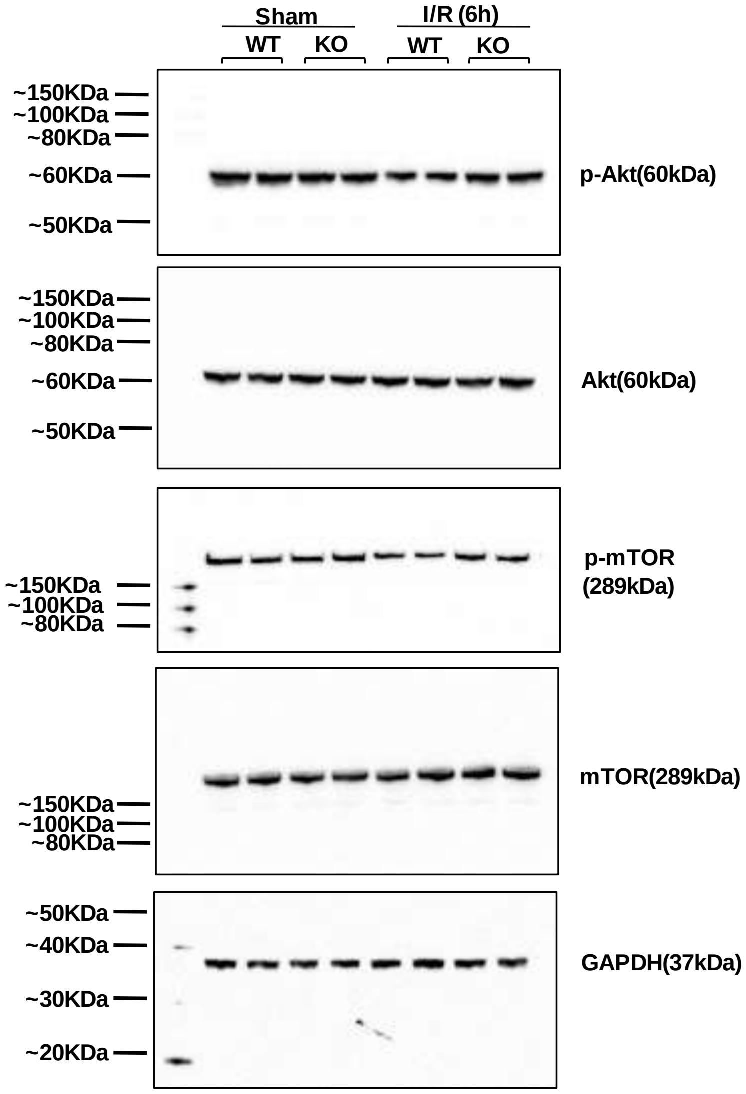

Full gels for Figure 5d

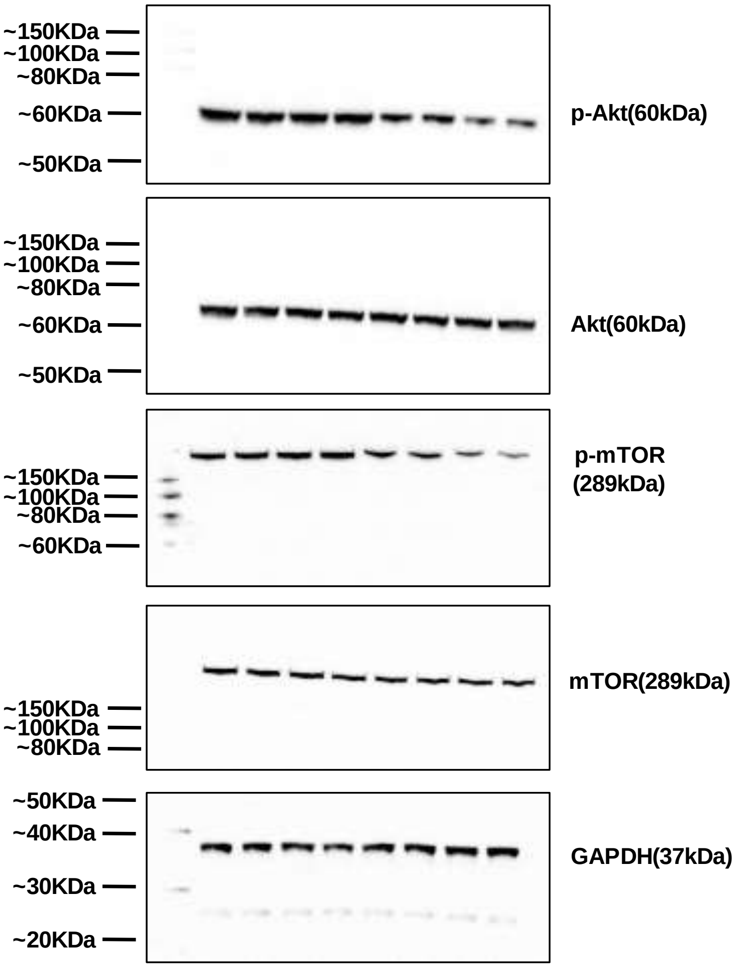

Full gels for Figure 6a

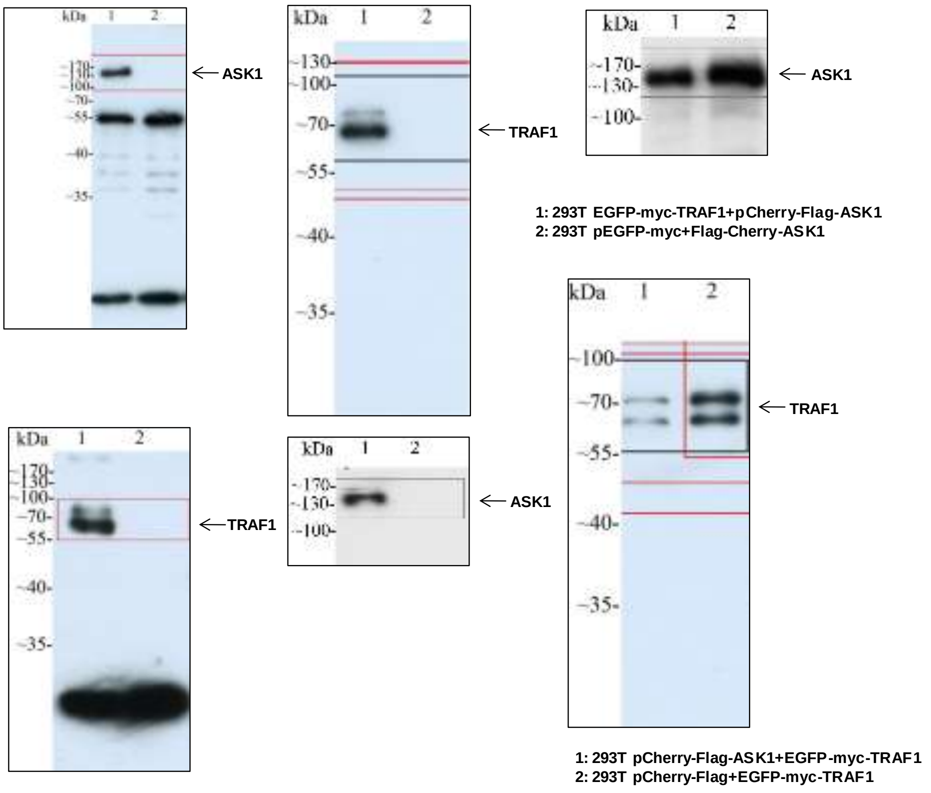

Full gels for Figure 6b

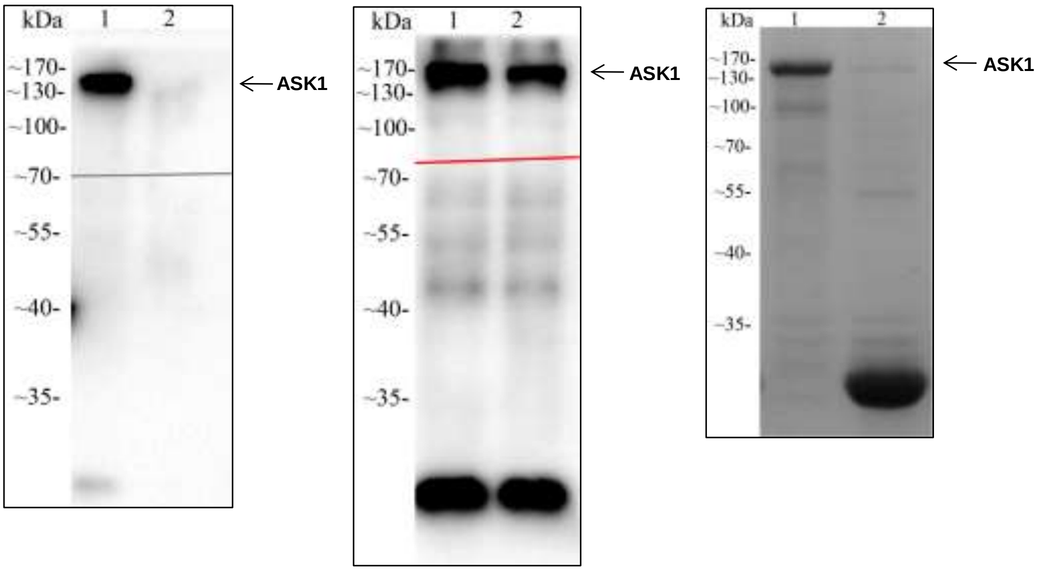

1: 293T GST-TRAF1+Cherry-Flag-ASK1
2: 293T GST-Cherry-Flag-ASK1

Full gels for Figure 6e

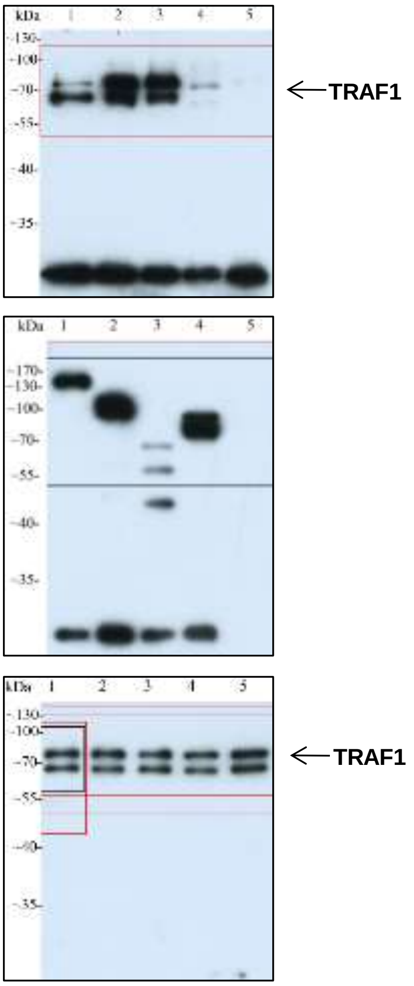

1: 293T EGFP-myc-TRAF1+Cherry-Flag-ASK1
2: 293T EGFP-myc-TRAF1+Cherry-Flag-ASK1-N
3: 293T EGFP-myc-TRAF1+Cherry-Flag-ASK1-K
4: 293T EGFP-myc-TRAF1+Cherry-Flag-ASK1-C
5: 293T EGFP-myc-TRAF1+Cherry-Flag

Full gels for Figure 6f

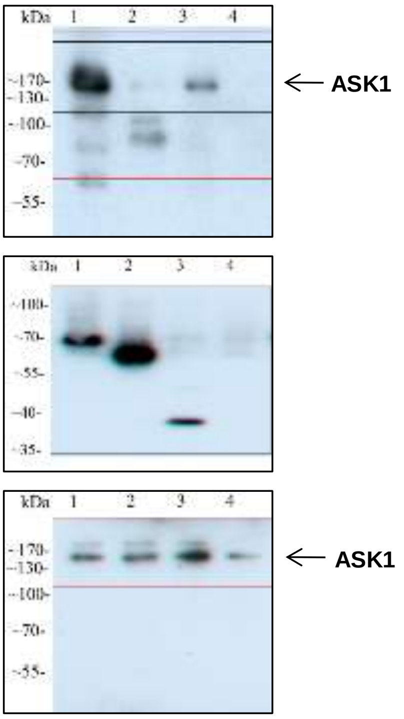

1: 293T EGFP-myc-TRAF1+Cherry-Flag-ASK1
2: 293T EGFP-myc-TRAF1-N+Cherry-Flag-ASK1
3: 293T EGFP-myc-TRAF1-C+Cherry-Flag-ASK1
4: 293T EGFP-myc-+Cherry-Flag-ASK1

Full gels for Figure 7e

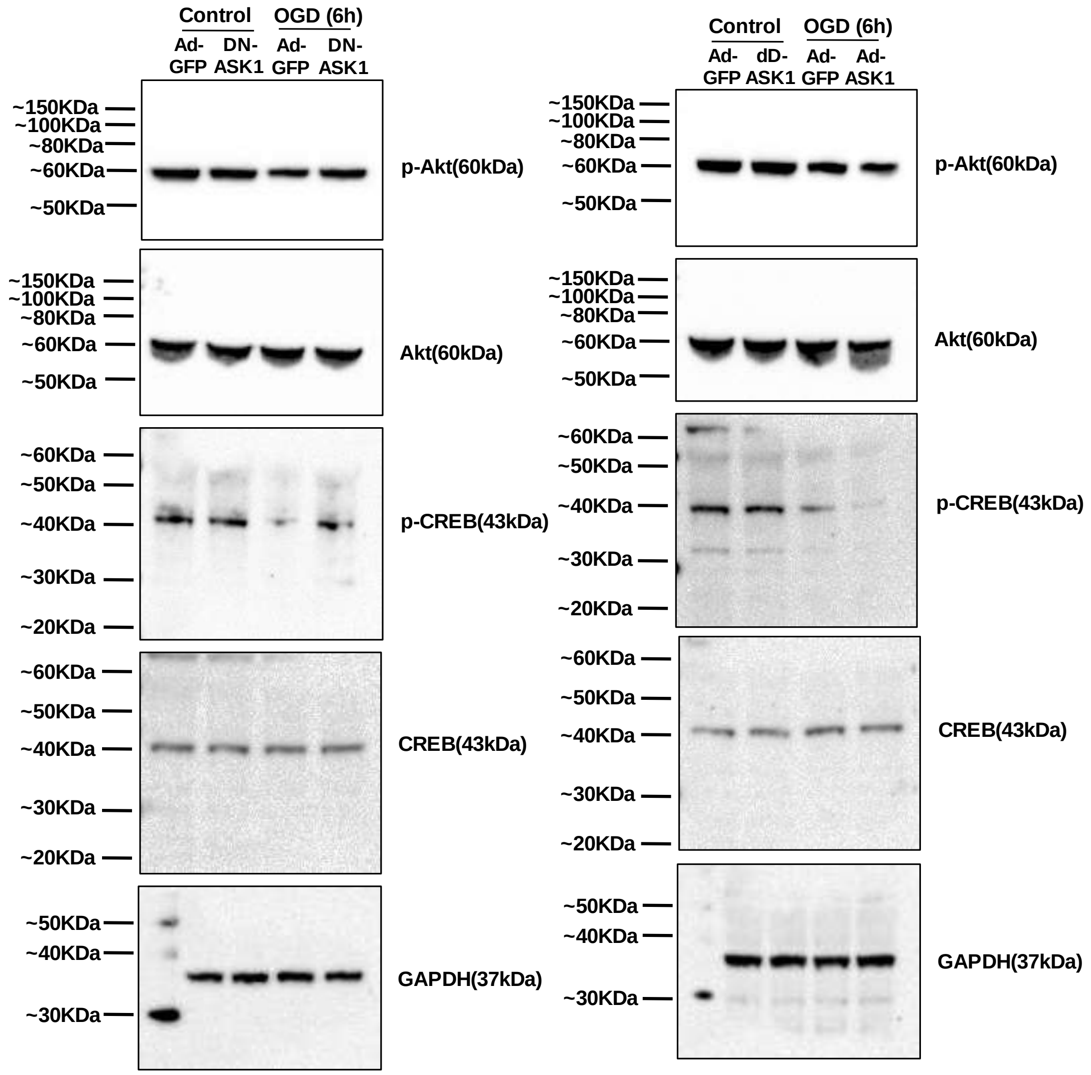

Full gels for Figure S2

b

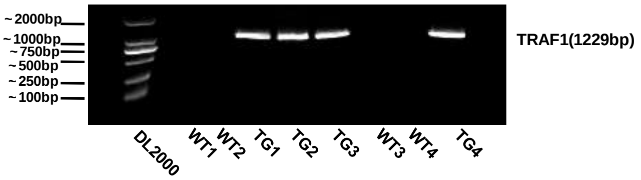

C

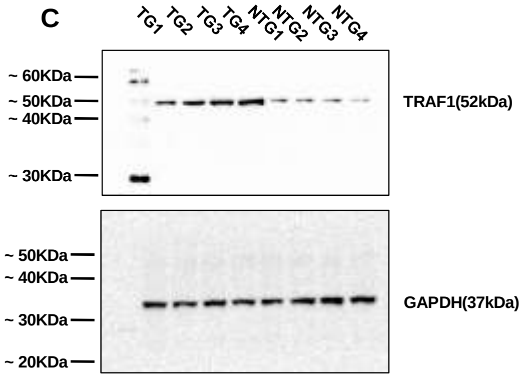

f

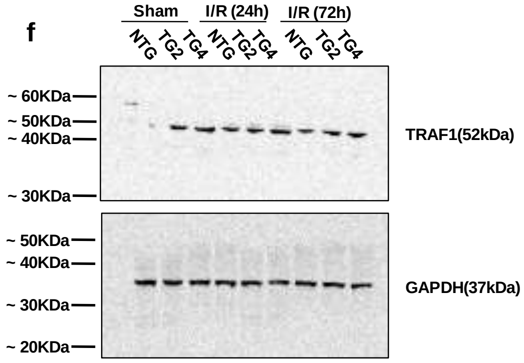

Full gels for Figure S3

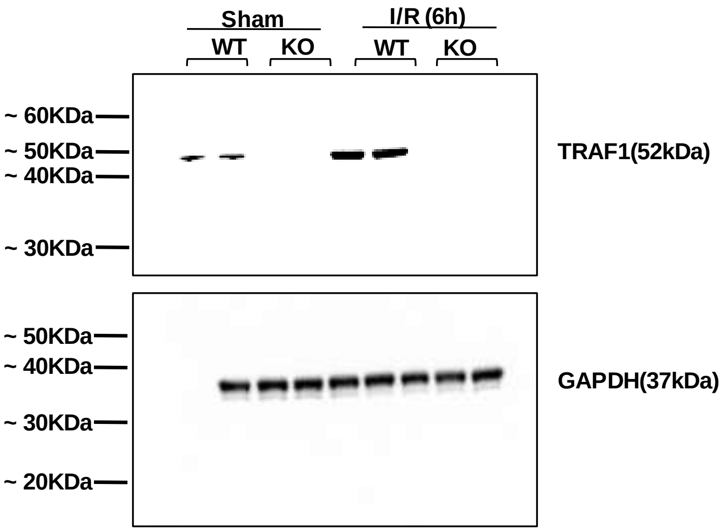

Full gels for Figure S4

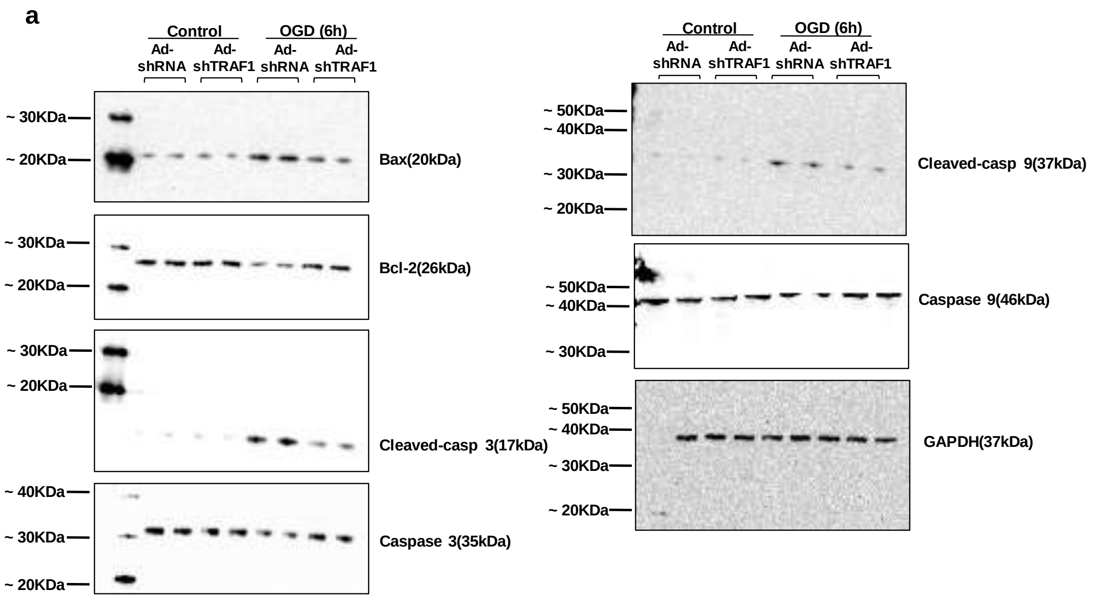

Full gels for Figure S4

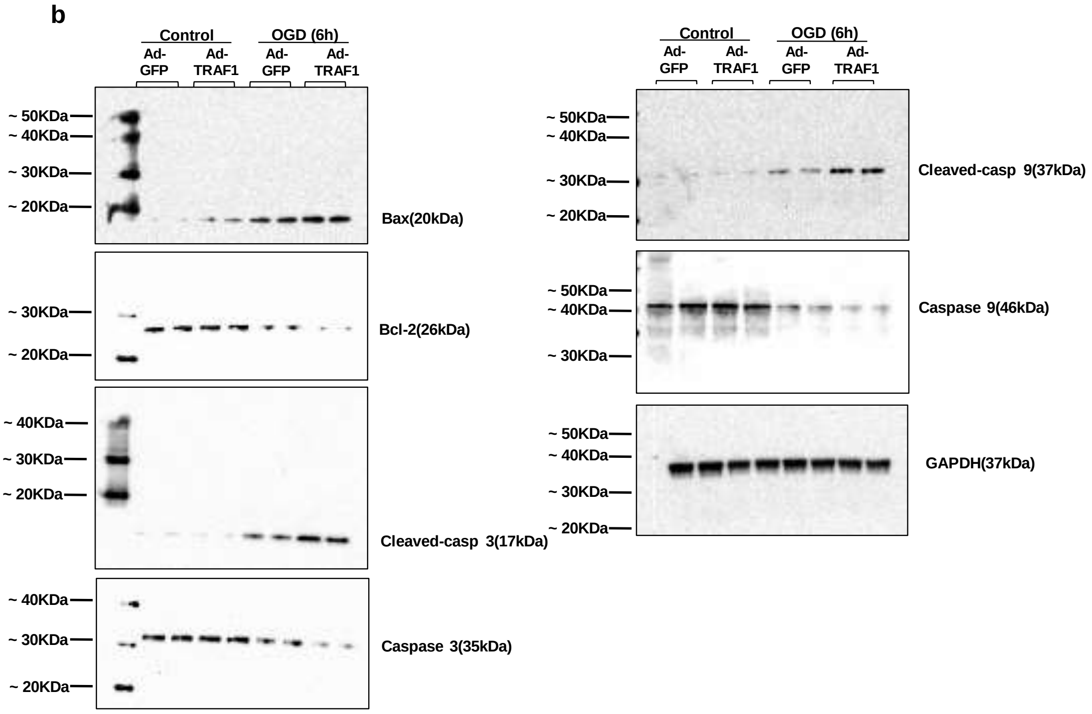

Full gels for Figure S5

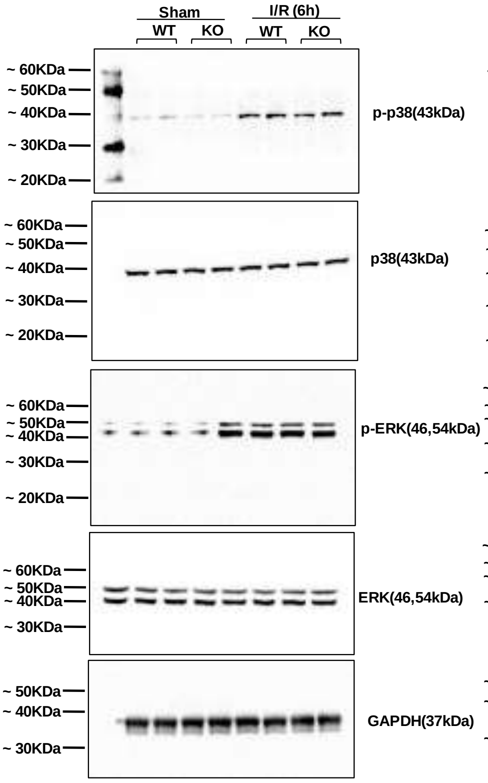

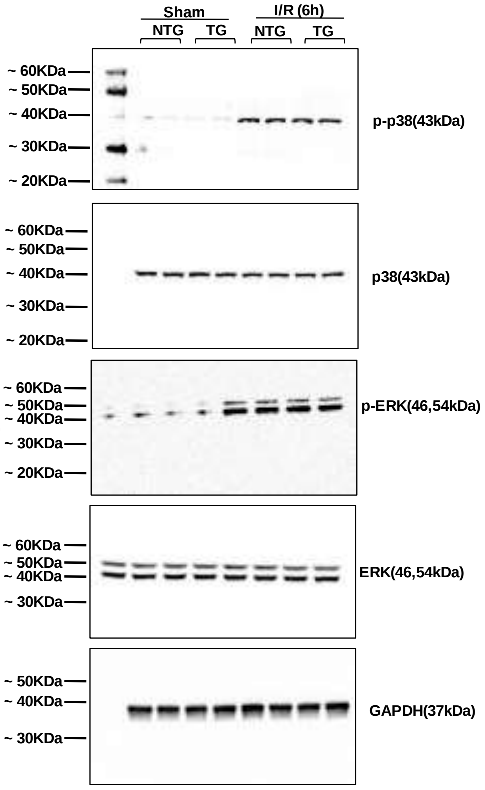

Full gels for Figure S6

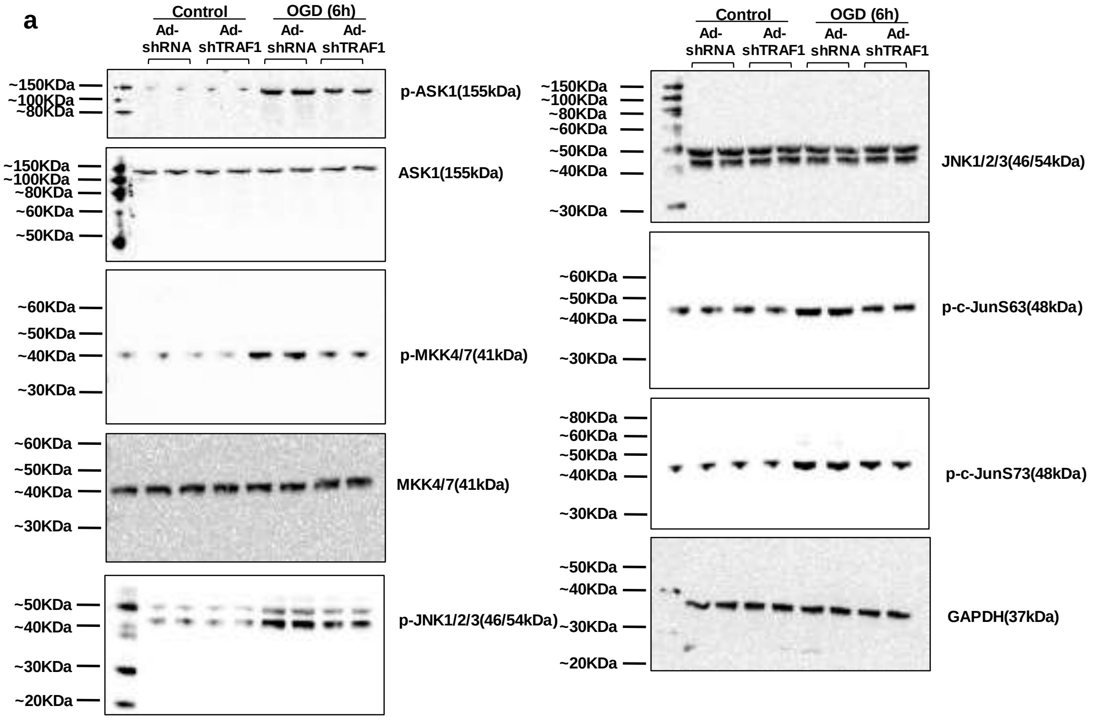

Full gels for Figure S6

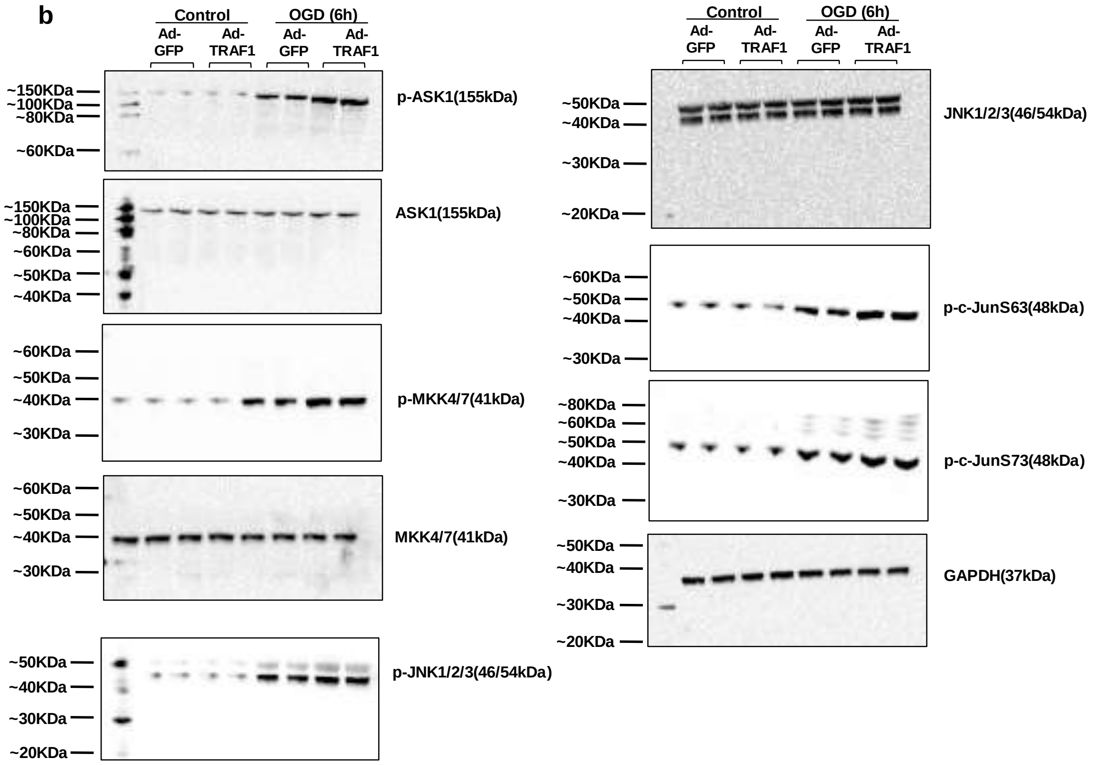

Full gels for Figure S7

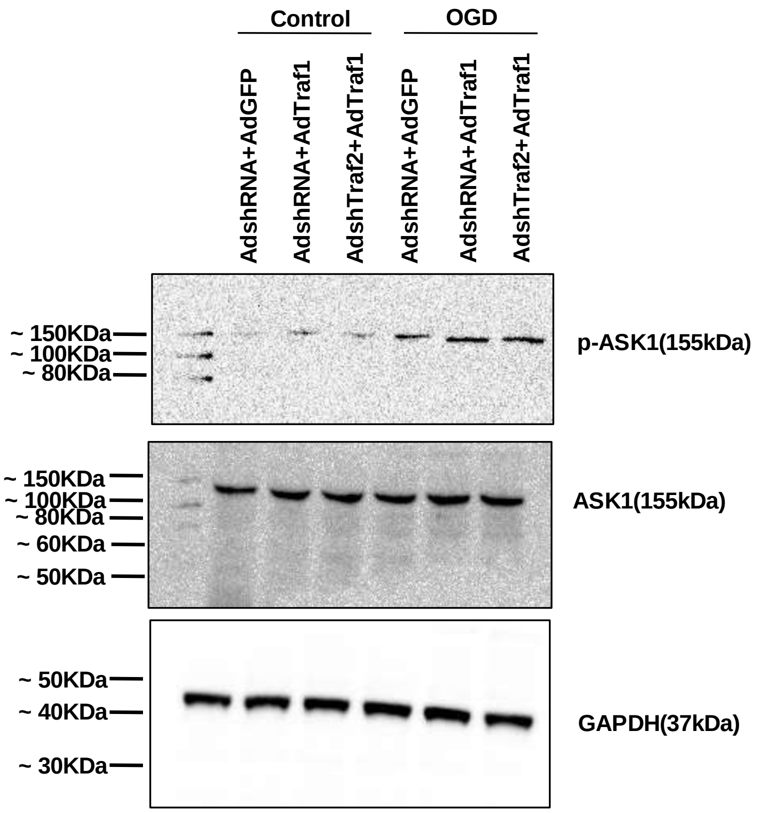

Full gels for Figure S8

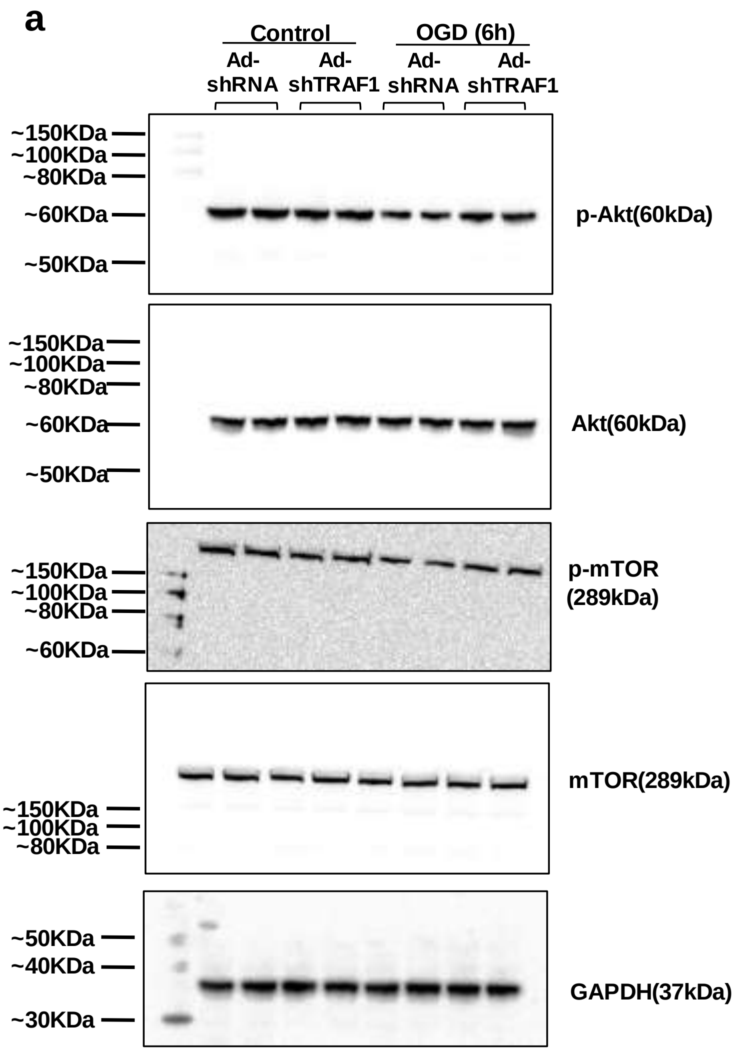

Full gels for Figure S8

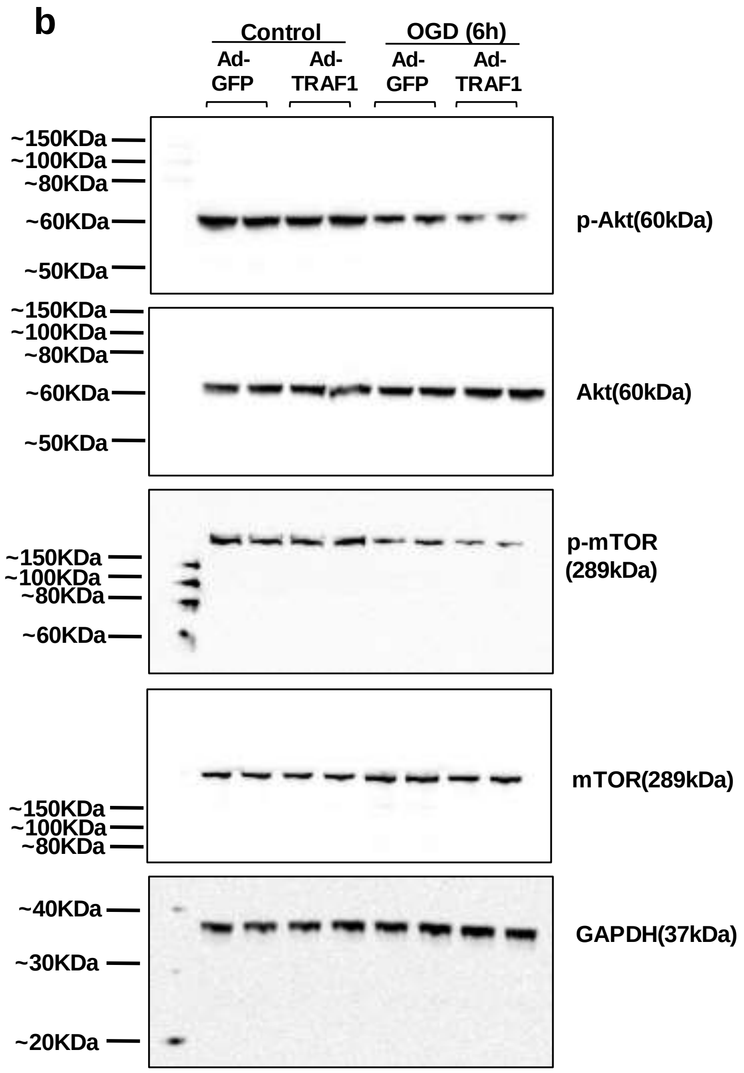

Full gels for Figure S9

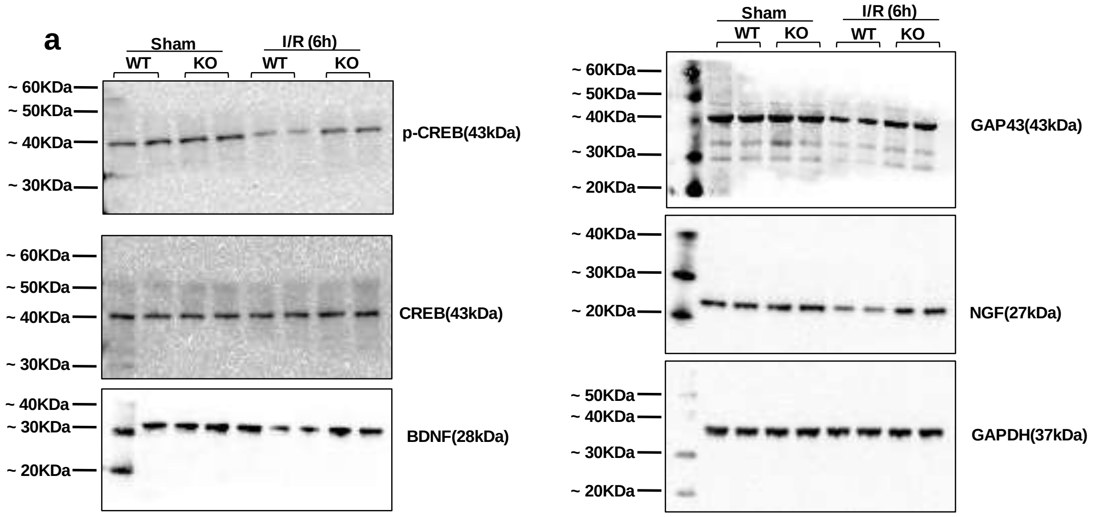

Full gels for Figure S9

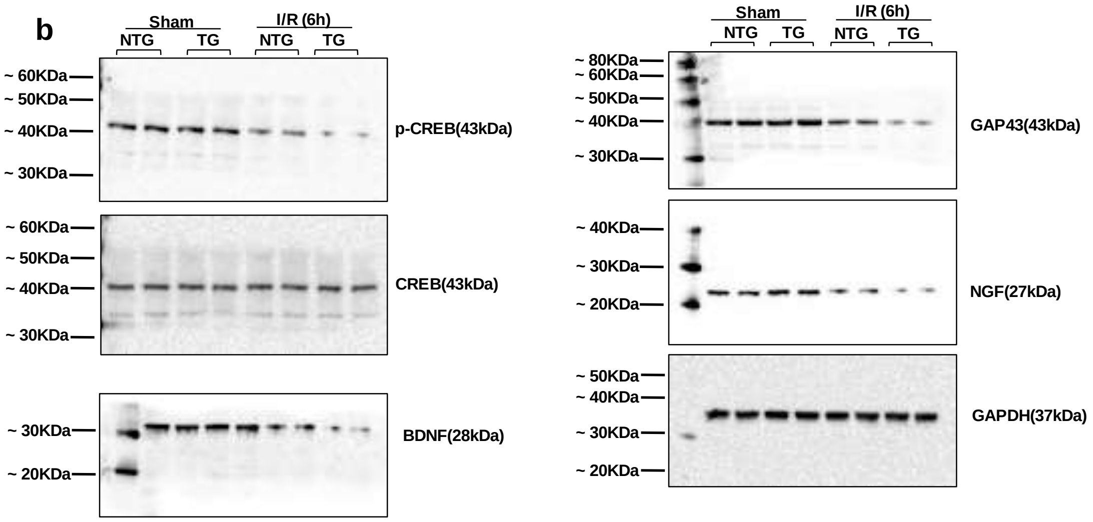

Full gels for Figure S10

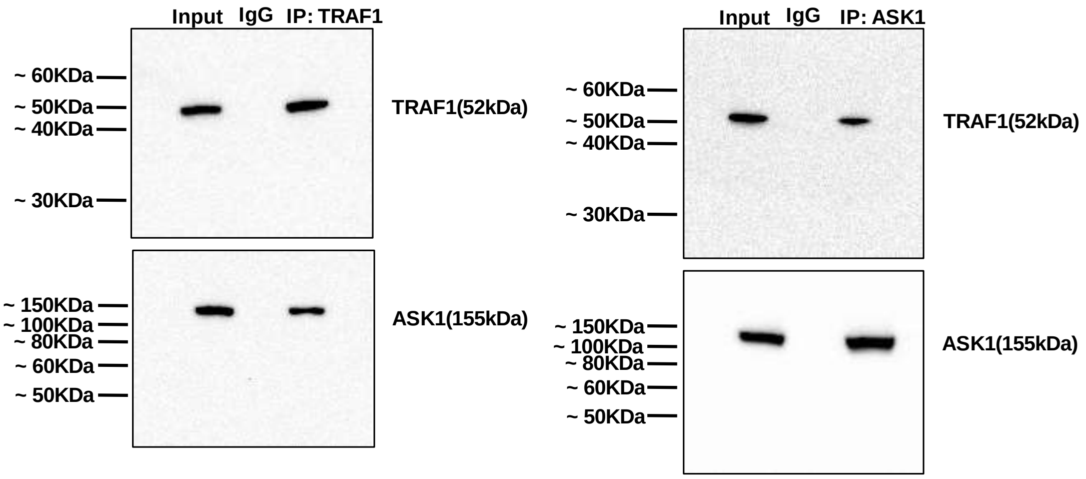

Full gels for Figure S11

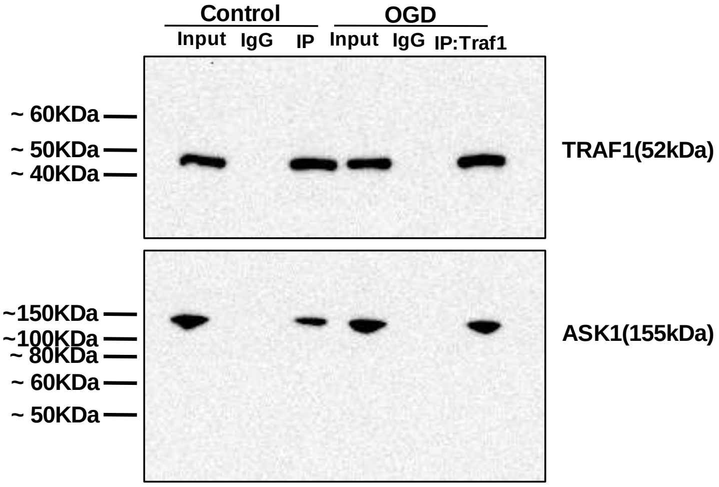

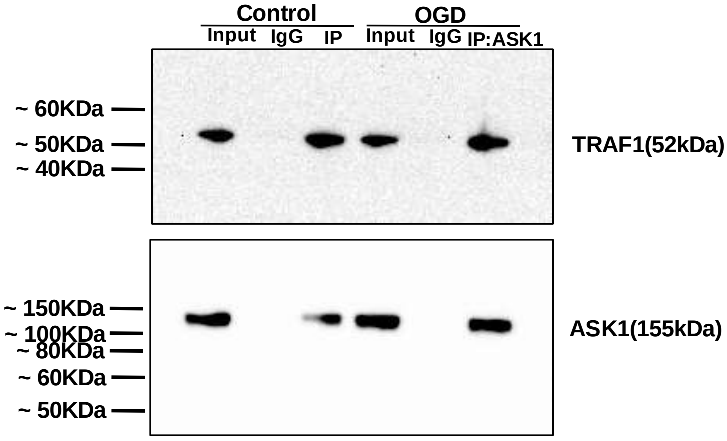

Supplementary Table S1

	WT	KO	NTG	TG2
**pH**	7.300±0.069	7.363±0.028	7.333±0.078	7.295±0.030
**pO_2 (mm Hg)**	106.00±3.37	116.25±22.01	105.25±10.01	127.00±23.26
**pCO_2 (mm Hg)**	37.75±5.50	28.25±8.88	31.00±8.29	29.14±10.42
**DBP (mm Hg)**	113.81±17.44	106.33±4.56	112.65±10.71	113.41±17.75
**SBP (mm Hg)**	137.38±18.93	136.8±9.09	138.4±9.09	139.12±18.67
**Heart rate (bpm)**	611.38±106.52	588.2±101.19	589.4±94.45	567.71±62.29
**Body weight (g)**	27.83±1.07	26.69±1.67	27.69±1.93	27.66±2.15

Supplementary Table S1. Physiological variables of experimental groups before surgery. Blood gas analysis, blood pressure, heart rate, and body weight were comparable between groups (n=8). WT: wild-type; KO: TRAF1 knockout; NTG: non-transgenic; TG: neuron-specific TRAF1 transgenic. SBP: systolic blood pressure; DBP: diastolic blood pressure. The data represent the mean ± s.d. Values are not significant between groups, as determined by unpaired Student's *t*-test.
